# Correction: Inference of past demography, dormancy and self-fertilization rates from whole genome sequence data

**DOI:** 10.1371/journal.pgen.1009504

**Published:** 2021-04-07

**Authors:** Thibaut Paul Patrick Sellinger, Diala Abu Awad, Markus Moest, Aurélien Tellier

The mutation and recombination rates reported throughout the article are incorrect by a factor of 2. The captions of Figs 1–4, Table 2, and S1–S19 Figs are incorrect. Additionally, the Y axes of Figs 5 and 6 and S20 Fig are shifted by a factor of 2. The authors provide corrected versions here. The correct rates were used for the simulations (S2 Appendix) and as such this error does not affect the conclusions of the study.

**Fig 1:**
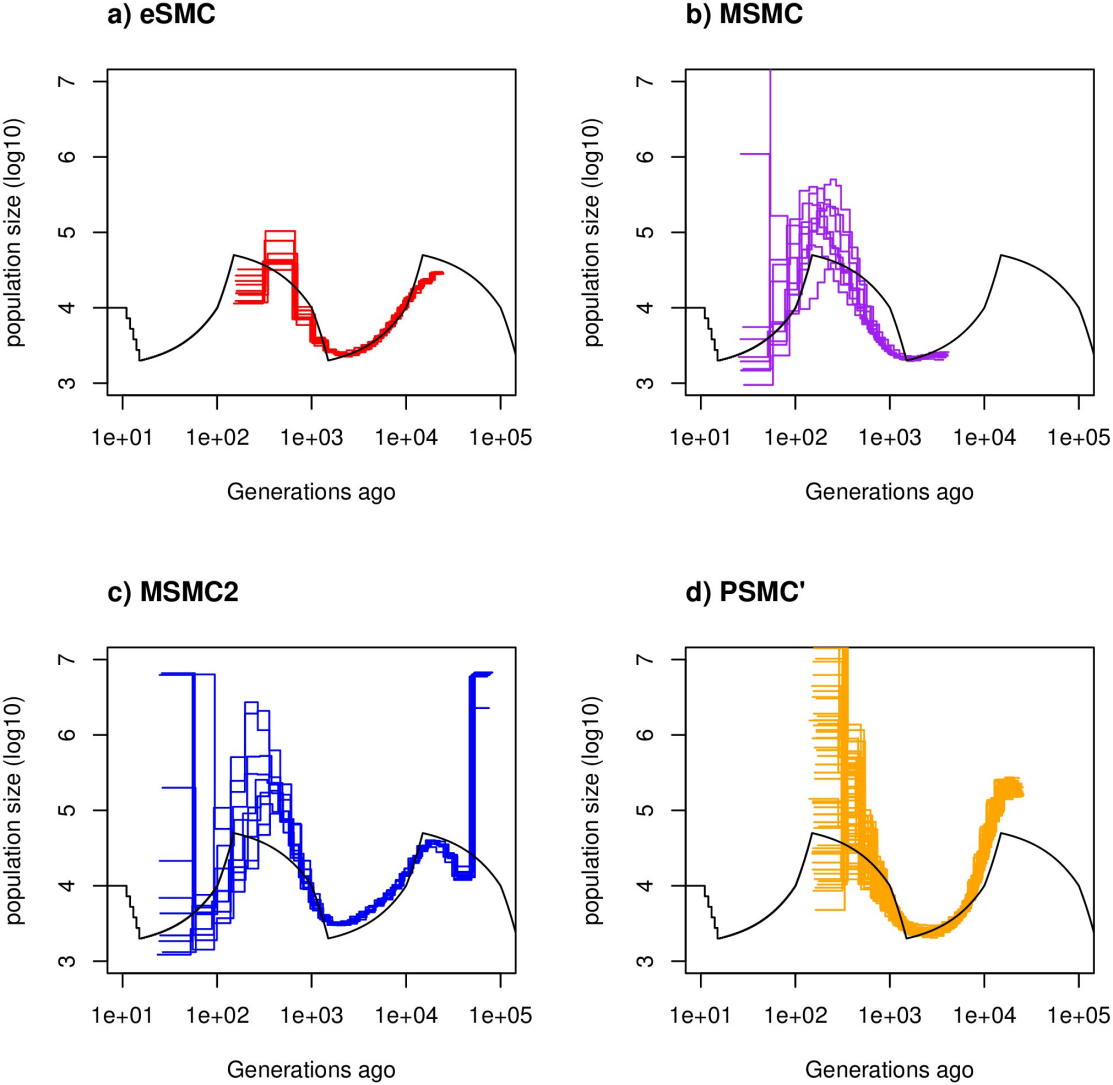
Estimated demographic history with no selfing or seed banking. Estimated demographic history using four simulated sequences of 30 Mb under a saw-tooth scenario with 10 replicates. Mutation and recombination rates (respectively *μ* and *r*) are set to 1.25 × 10^−8^ per generation per bp. Therefore $\frac{\rho }{\theta }=\frac{r}{\mu }=1$. The simulated demographic history is represented in black. a) Demographic history estimated by eSMC (red). b) Demographic history estimated by MSMC (purple). c) Demographic history estimated by MSMC2 (blue). d) Demographic history estimated by PSMC’ (orange).

**Fig 2:**
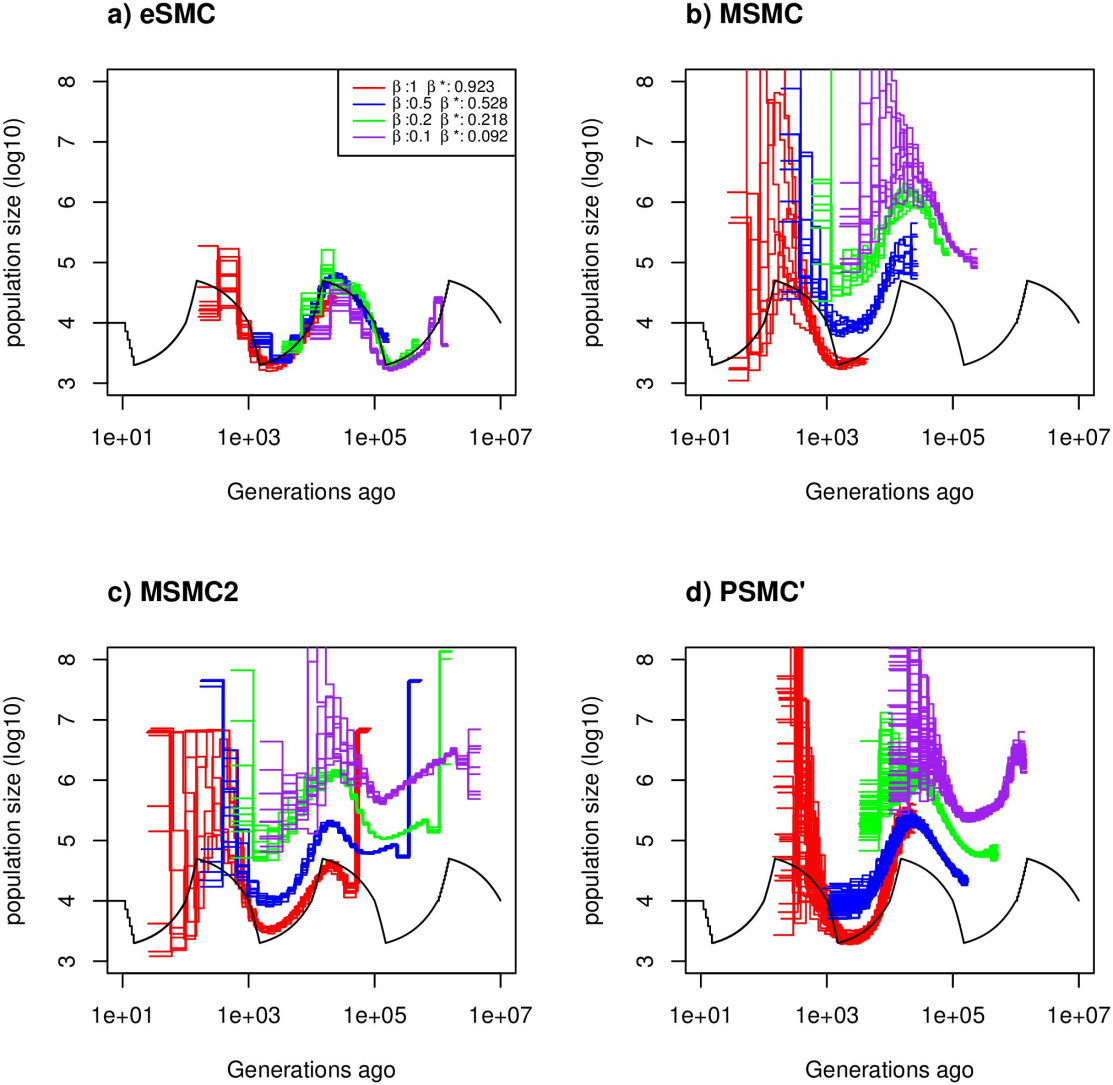
Estimated demographic history with seed banking. Estimated demographic history using four simulated sequences of 10 Mb and ten replicates under a saw-tooth demographic scenario (black). The mutation and recombination rates are set to 1.25 × 10^−8^ per generation per bp. Therefore $\frac{r}{\mu }=1$. We simulate under four different germination rates *β* = 1 (red), 0.5 (blue), 0.2 (green) and 0.1 (purple), hence we respectively have $\frac{\rho }{\theta }=1$, 0.5, 0.2 and 0.1. The demographic history is estimated using a) eSMC, b) MSMC, c) MSMC2 and d) PSMC’. β* represents the estimated germination rate by eSMC.

**Fig 3:**
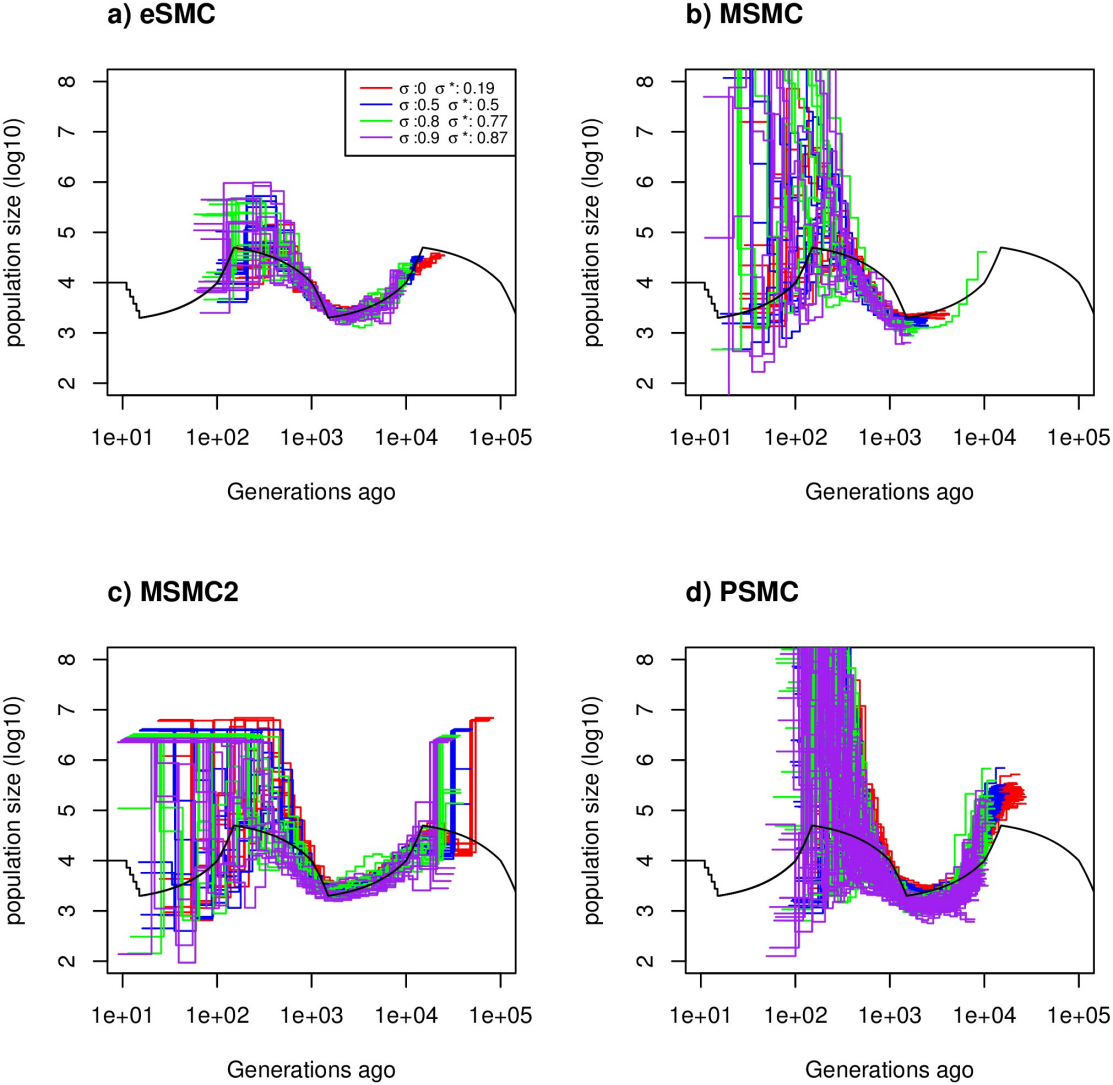
Estimated demographic history with selfing. Estimated demographic history using four simulated sequences of 10 Mb and ten replicates under a saw-tooth demographic scenario (black). The mutation and recombination rates are set to 1.25 × 10^−8^ per generation per bp, and simulations were run for four different self-fertilization rates (*σ* = 0 (red), 0.5 (blue), 0.8 (green) and 0.9 (purple)), and as $\frac{r}{\mu }=1$, this gives $\frac{\rho }{\theta }=1$, 0.6667, 0.333 and 0.182 respectively. The demographic history is estimated using a) eSMC, b) MSMC, c) MSMC2 and d) PSMC’. σ* represents the self-fertilization rate estimated by eSMC.

**Fig 4:**
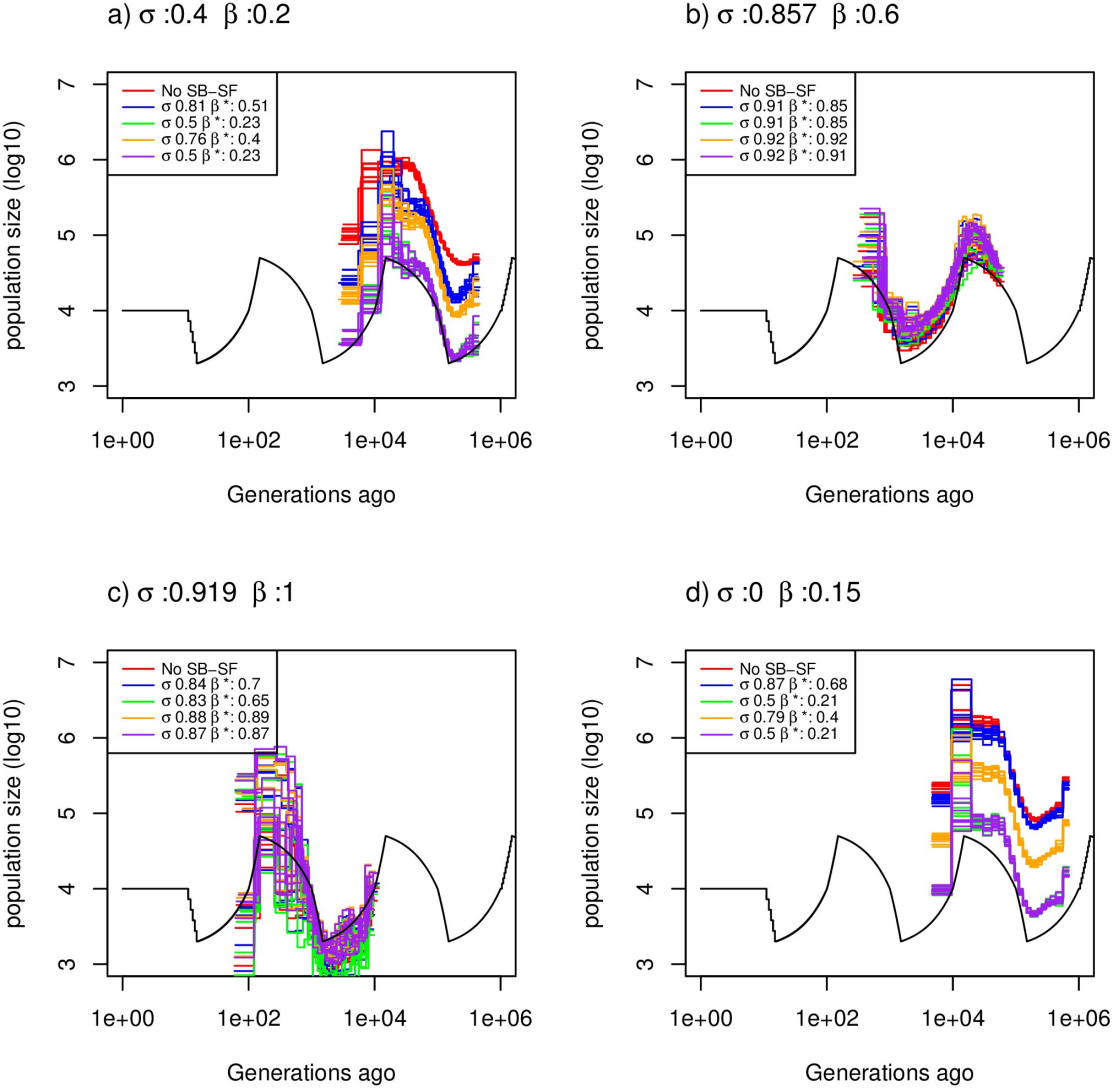
Estimated demographic history with selfing and seed banking. Demographic history estimated by eSMC for ten replicates using four simulated sequences of 10 Mb under a saw-tooth demographic scenario and four different combinations of germination (*β*) and self-fertilization (*σ*) rates but resulting in the same $\frac{\rho }{\theta }$. Mutation and recombination rates are set to 1.25 × 10^−8^ per generation per bp, giving $\frac{r}{\mu }=1$. The four combinations are: a) *σ* = 0.4 and *β* = 0.25, b) *σ* = 0.75 and *β* = 0.6, c) *σ* = 0.85 and *β* = 1 and d) *σ* = 0 and *β* = 0.15. Hence, for each scenario $\frac{\rho }{\theta }=0.15$. For each combination of *β* and *σ*, eSMC was launched with five different prior settings: ignoring seed-banks and self-fertilization (red), accounting for seed-banks and self-fertilization but without setting priors (blue), accounting for seed-banks and self-fertilization with a prior set only for the self-fertilization rate (green), only for the germination rate (orange) or for both (purple). σ* and β* respectively represent the estimated self-fertilization and germination rate.

**Fig 5:**
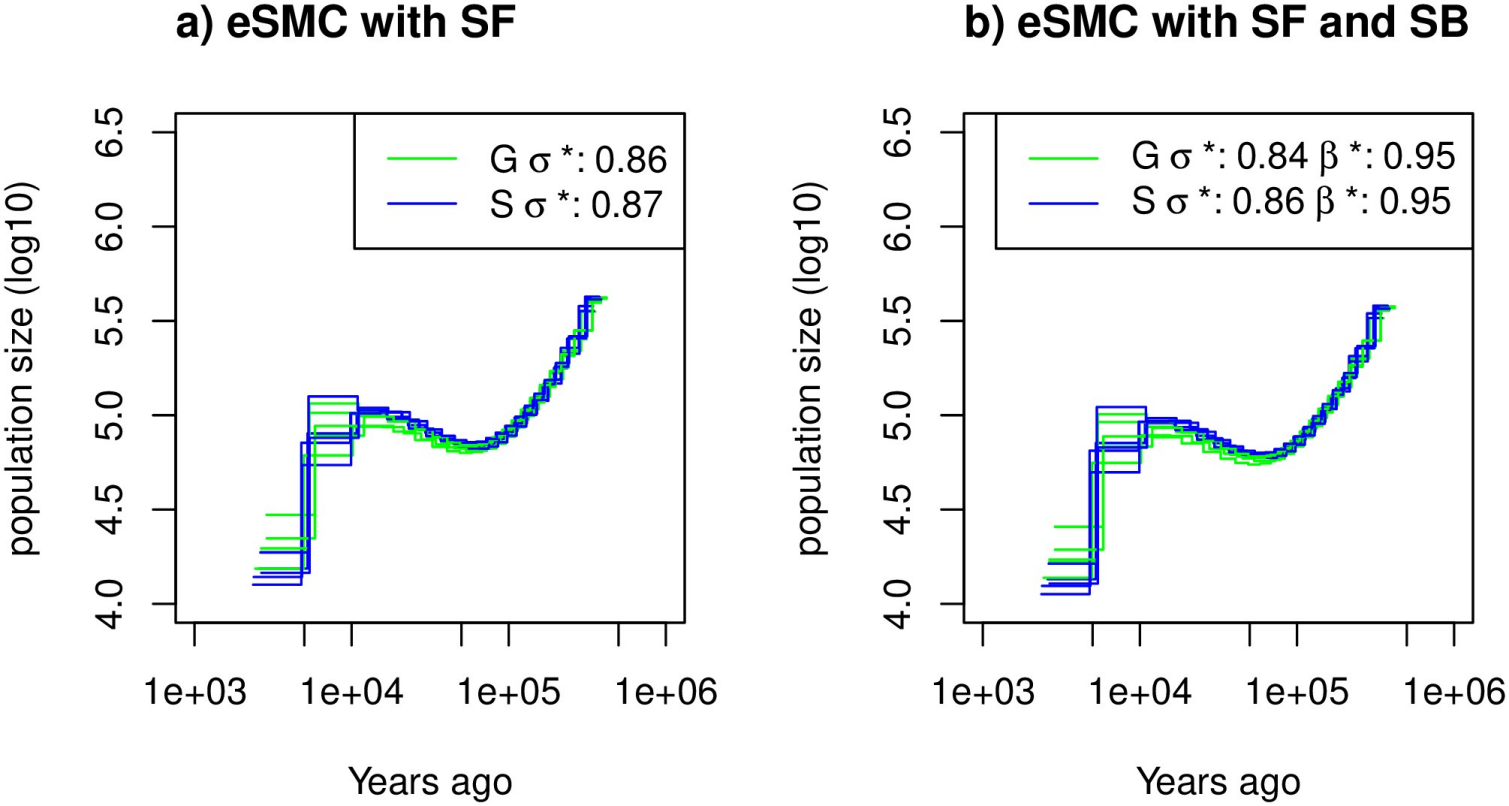
Estimated demographic history of *Arabidopsis thalinana*. Demographic history of two European (Sweden (S, blue) and German (G, green)) populations of *A*. *thaliana* estimated using eSMC: a) accounting only for selfing (*σ* is a variable and *β* = 1) and b) accounting simultaneously for selfing and seed-banking (*σ* bounded between 0.5 and 0.99 and *β* bounded between 0.5 and 1). Mutation rate is set to 7 × 10^−9^ per generation per bp and recombination respectively set for chromosome 1 to 5 to 3.4 × 10^−8^, 3.6 × 10^−8^, 3.5 × 10^−8^, 3.8 × 10^−8^, 3.6 × 10^−8^) per generation per bp. σ* and β* respectively represent the estimated self-fertilization and germination rates.

**Fig 6:**
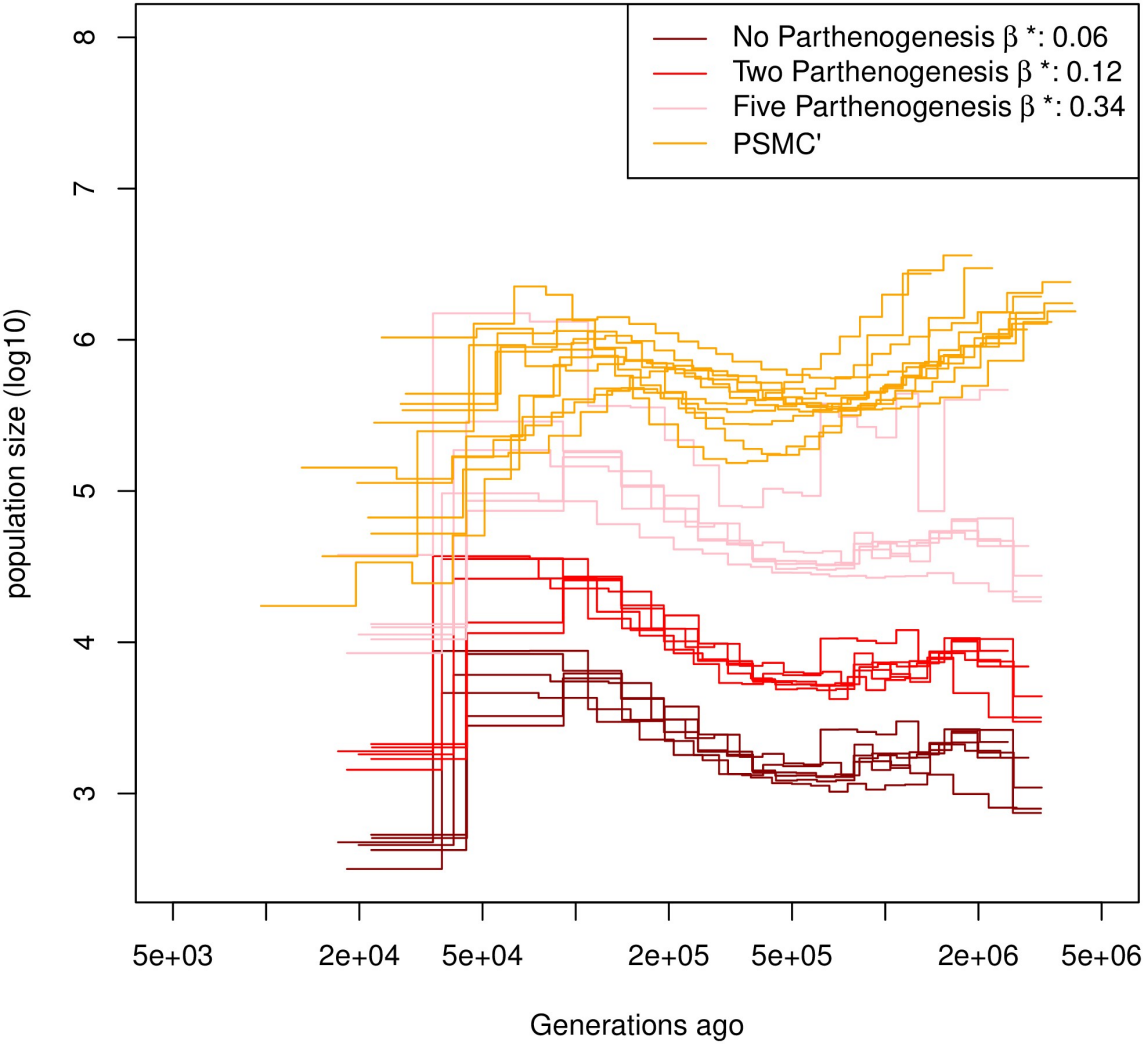
Estimated demographic history of *Daphnia pulex*. Demographic history estimated by eSMC on six individuals of *D*. *pulex* accounting for egg-banks (*β* is a variable and *σ* = 0). Different assumptions concerning the number of parthenogenetic cycles before the production of the dormant egg are made: Five cycles (pink), two cycles (red) and no parthenogenesis (dark red). A subset of demographic history estimated by PSMC’ are plotted in orange. Mutation and recombination rates are respectively set to 4.33 × 10^−9^ and $\frac{8\times 10^{-8}}{n_{p}}$ per generation per bp, where *n*_*p*_ is the number of reproductive cycles per year, parthenogenetic and sexual.

**Table 2:** Calculation times of eSMC on simulated data under the “saw-tooth” demographic scenario with mutation and recombination rate set to 1.25 × 10^−8^ per generation per bp. Results are in minutes given the sequence length and the number of haplotypes.

Sequence length (Mb)	2 Haplotypes	4 Haplotypes	10 Haplotypes
1 Mb	12	13	14
10 Mb	14	16	31
30 Mb	19	25	75

There are several errors in the Simulation results subsection of the Results as listed below.

In the Convergence property in the absence of seed-banks and self-fertilization subheading, there are errors in the first sentence of the fourth paragraph. The correct sentence is: We now assume $\frac{\rho }{\theta }=\frac{r}{\mu }=5$, with the mutation and recombination rate respectively set to 1.25 × 10^−8^ and 6.25 × 10^−8^ per generation per nucleotide.

In the Convergence property with dormancy (seed- or egg-banks) subheading of the Simulation results subsection of the Results, there is an error in the first sentence of the first paragraph. The correct sentence is: Using eSMC on sequences simulated under the “saw-tooth” scenario in the presence of seed-banks (mutation and recombination rates are set to 1.25 × 10^−8^ per generation per bp, (Fig 2), we obtain an accurate estimation of the demography (*χ*_*t*_) and of the germination rates (*β*). There is also an error in the last sentence of the first paragraph. The correct sentence is: Therefore when the molecular mutation and recombination are set to 2.5 × 10^−9^ per generation per bp, better fits are obtained (S12 Fig).

In the Convergence property with dormancy (seed- or egg-banks) subheading, there are several errors in the second paragraph. The correct paragraph is: For simpler demographic scenarios (constant population size, bottleneck, expansion and decrease, see S13 Fig) and *μ* = *r* = 1.25 × 10^−8^ per generation per bp, the germination rate and the demographic histories estimated by eSMC are accurate for most of the demographic scenarios considered, except in the case of a bottleneck scenario (as expected from previous results). In presence of strong seed-banks (*β* = 0.2 or 0.1) there are biases in estimations of the far past. Once again, this tendency disappears when the molecular mutation and recombination rates per site are lowered so as not to violate the infinite site model (*μ* and *r* = 2.5 × 10^−9^ per generation per bp, see S14 Fig).

In the Convergence property with self-fertilization subheading, there is an error in the first sentence of the first paragraph. The correct sentence is: Under the “saw-tooth” scenario with different rates of self-fertilization *σ*, with mutation and recombination rates set to 1.25 × 10^−8^ per generation per bp ($\frac{r}{\mu }=1$), for four different self-fertilization rates *σ* = 0 (no self-fertilization), 0.5 (50% selfing), 0.8 (80% selfing) and 0.9 (90% selfing), we estimate the self-fertilization rate respectively at 0.19, 0.5, 0.77 and 0.87 (Fig 3). There are also errors in the fifth sentence of the first paragraph. The correct sentence is: When the mutation rate is set to 1.25 × 10^−8^ per generation per bp and the recombination rate to 6.25 × 10^−8^ per generation per nucleotide ($\frac{r}{\mu }=5$), the self-fertilization rate is overestimated for small values of *σ* (S15 Fig), but well estimated for higher values of *σ*.

In the Convergence property with both dormancy and self-fertilization subheading, there is an error in the first sentence of the first paragraph. The correct sentence is: Here we test different combinations of seed/egg-banks and self-fertilization rates that result in the same ratio $\frac{\rho }{\theta }=0.15$, with $\frac{r}{\mu }=1$ (setting *μ* = *r* = 1.25 × 10^−8^ per generation per bp). There is also an error in the eleventh sentence of the first paragraph. The correct sentence is: We also test how recombination can influence the output of these models, notably by taking a higher recombination rate (8.335 × 10^−8^ per site per generation), more representative of the high recombination to mutation ratio observed in some species (notably *D*. *pulex* and *A*. *thaliana* [4, 45]).

There is a minor error in S2 Appendix. The command lines for S8, 10, and 11 Fig are incorrect. Please view the correct S2 Appendix below.

## Supporting information

S2 Appendix Command lines. Contains all scrm command line to reproduce all tested scenario.
(PDF)

S1 FigEstimated demographic history in absence of selfing or seed banking using sequences of 10 Mb.Estimated demographic history using four simulated sequences of 10 Mb under a saw-tooth demographic scenario with 10 replicates. Mutation and recombination rate are set to 1.25 × 10^−8^ per generation per bp. Therefore $\frac{\rho }{\theta }=\frac{r}{\mu }=1$. The simulated demographic history is represented in black. a) Demographic history estimated by eSMC (red). b) Demographic history estimated by MSMC (purple). c) Demographic history estimated by MSMC2 (blue). d) Demographic history estimated by PSMC’ (orange).(TIF)

S2 FigEstimated demographic history in absence of selfing or seed banking using sequences of 10 Mb when all method have same discretization of the population size as eSMC).Estimated demographic history using four simulated sequences of 10 Mb under a sawtooth demographic scenario with 10 replicates. Mutation and recombination rate are set to 1.25 × 10^−8^ per generation per bp. Therefore $\frac{\rho }{\theta }=\frac{r}{\mu }=1$. The simulated demographic history is represented in black. a) Demographic history estimated by eSMC (red). b) Demographic history estimated by MSMC (purple). c) Demographic history estimated by MSMC2 (blue). d) Demographic history estimated by PSMC’ (orange).(TIF)

S3 FigEstimated demographic history in absence of selfing or seed banking using sequences of 1 Mb.Estimated demographic history using four simulated sequences of 1 Mb under a saw-tooth scenario with 10 replicates. Mutation and recombination rate are set to 1.25 × 10^−8^ per generation per bp. Therefore $\frac{\rho }{\theta }=\frac{r}{\mu }=1$. The simulated demographic history is represented in black. a) Demographic history estimated by eSMC (red). b) Demographic history estimated by MSMC (purple). c) Demographic history estimated by MSMC2 (blue). d) Demographic history estimated by PSMC’ (orange).(TIF)

S4 FigEstimated demographic history using eSMC in four simple demographic scenarios.Estimated demographic history using four simulated sequences of 10 Mb under 4 different demographic scenarios with 10 replicates. Mutation and recombination rate are set to 1.25 × 10^−8^ per generation per bp. Therefore $\frac{\rho }{\theta }=\frac{r}{\mu }=1$. The simulated demographic history is represented in black. a) Demographic history simulated under a constant population size. b) Demographic history simulated under a bottleneck. c) Demographic history simulated under an expansion. d) Demographic history simulated under a decrease. Demographic history estimated by eSMC is in red.(TIF)

S5 FigEstimated demographic history using PSMC’ in four simple demographic scenarios.Estimated demographic history using four simulated sequences of 10 Mb under 4 different demographic scenarios with 10 replicates. Mutation and recombination rate are set to 1.25 × 10^−8^ per generation per bp. Therefore $\frac{\rho }{\theta }=\frac{r}{\mu }=1$. The simulated demographic history is represented in black. a) Demographic history simulated under a constant population size. b) Demographic history simulated under a bottleneck. c) Demographic history simulated under an expansion. d) Demographic history simulated under a decrease. Demographic history estimated by PSMC’ is in orange.(TIF)

S6 FigEstimated demographic history using MSMC in four simple demographic scenarios.Estimated demographic history using four simulated sequences of 10 Mb under 4 different demographic scenarios with 10 replicates. Mutation and recombination rate are set to 1.25 × 10^−8^ per generation per bp. Therefore $\frac{\rho }{\theta }=\frac{r}{\mu }=1$. The simulated demographic history is represented in black. a) Demographic history simulated under a constant population size. b) Demographic history simulated under a bottleneck. c) Demographic history simulated under an expansion. d) Demographic history simulated under a decrease. Demographic history estimated by MSMC is in purple.(TIF)

S7 FigEstimated demographic history using MSMC2 in four simple demographic scenarios.Estimated demographic history using four simulated sequences of 10 Mb under 4 different demographic scenarios with 10 replicates. Mutation and recombination rate are set to 1.25 × 10^−8^ per generation per bp. Therefore $\frac{\rho }{\theta }=\frac{r}{\mu }=1$. The simulated demographic history is represented in black. a) Demographic history simulated under a constant population size. b) Demographic history simulated under a bottleneck. c) Demographic history simulated under an expansion. d) Demographic history simulated under a decrease. Demographic history estimated by MSMC2 is in blue.(TIF)

S8 FigEstimated demographic history under $\frac{r}{\mu }=5$.**Results are obtained by fixing recombination rate to real value**. Estimated demographic history using four simulated sequences of 10 Mb under a saw-tooth scenario with 10 replicates. Mutation and recombination rate are set to 1.25 × 10^−8^ and 6.25 × 10^−8^ per generation per bp. Therefore $\frac{\rho }{\theta }=\frac{r}{\mu }=5$. The simulated demographic history is represented in black. a) Demographic history estimated by eSMC (red). b) Demographic history estimated by MSMC (purple). c) Demographic history estimated by MSMC2 (blue). d) Demographic history estimated by PSMC’ (orange).(TIF)

S9 FigEstimated demographic history under $\frac{r}{\mu }=100$.**Results are obtained by fixing recombination rate to real value**. Estimated demographic history using four simulated sequences of 10 Mb under a saw-tooth scenario with 10 replicates. Mutation and recombination rate are set to 1.25 × 10^−8^ and 1.25 × 10^−6^ per generation per bp. Therefore $\frac{\rho }{\theta }=\frac{r}{\mu }=100$. The simulated demographic history is represented in black. a) Demographic history estimated by eSMC (red). b) Demographic history estimated by MSMC (purple). c) Demographic history estimated by MSMC2 (blue). d) Demographic history estimated by PSMC’ (orange).(TIF)

S10 FigEstimated demographic history under $\frac{r}{\mu }=5$ with initial value $\frac{r}{\mu }=5$.Results are obtained by estimating recombination rate with initial value equal to mutation rate ($\frac{\rho }{\theta }=5$). Estimated demographic history using four simulated sequences of 10 Mb under a saw-tooth scenario with 10 replicates. Mutation and recombination rate are set to 1.25 × 10^−8^ and 6.25 × 10^−8^ per generation per bp. Therefore $\frac{\rho }{\theta }=\frac{r}{\mu }=5$. The simulated demographic history is represented in black. a) Demographic history estimated by eSMC (red). b) Demographic history estimated by MSMC (purple). c) Demographic history estimated by MSMC2 (blue). d) Demographic history estimated by PSMC’ (orange).(TIF)

S11 FigEstimated demographic history under $\frac{r}{\mu }=5$ with initial value $\frac{r}{\mu }=1$.Results are obtained by estimating recombination rate with initial value equal to mutation rate ($\frac{\rho }{\theta }=1$). Estimated demographic history using four simulated sequences of 10 Mb under a saw-tooth scenario with 10 replicates. Mutation and recombination rate are set to 1.25 × 10^−8^ and 6.25 × 10^−8^ per generation per bp. Therefore $\frac{\rho }{\theta }=\frac{r}{\mu }=5$. The simulated demographic history is represented in black. a) Demographic history estimated by eSMC (red). b) Demographic history estimated by MSMC (purple). c) Demographic history estimated by MSMC2 (blue). d) Demographic history estimated by PSMC’ (orange).(TIF)

S12 FigEstimated demographic history with seed banking and μ = 2.5 × 10^−9^.Estimated demographic history using four simulated sequences of 10 Mb and ten replicates under a sawtooth demographic scenario (black). Simulation were done under four different germination rate *β* (1,0.5,0.2 and 0.1). The mutation and recombination rates are set to 2.5 × 10^−9^ per generation per bp. Therefore $\frac{r}{\mu }=1$ and respectively $\frac{\rho }{\theta }=1$, $\frac{\rho }{\theta }=0.5$, $\frac{\rho }{\theta }=0.2$ and $\frac{\rho }{\theta }=0.1$. Estimated demographic history are represented for all tested germination rate, *β* = 1 (red), 0.5 (blue), 0.2 (green) and 0.1 (purple). The demographic history is estimated using a) eSMC where β* equal the estimated germination rate, b) MSMC, c) MSMC2 and d) PSMC’.(TIF)

S13 FigEstimated demographic history in four simple demographic scenarios with seed banking.Estimated demographic history using four simulated sequences of 10 Mb under four different demographic scenarios with 10 replicates. Mutation and recombination rate are set to 1.25 × 10^−8^ per generation per bp. Simulation were done under four different germination rates *β*. We have *β* = 1 (red), 0.5 (blue), 0.2 (green) and 0.1 (purple). Therefore $\frac{r}{\mu }=1$ and respectively $\frac{\rho }{\theta }=1$, $\frac{\rho }{\theta }=0.5$, $\frac{\rho }{\theta }=0.2$ and $\frac{\rho }{\theta }=0.1$. The simulated demographic history is represented in black. a) Demographic history simulated under a constant population size. b) Demographic history simulated under a bottleneck. c) Demographic history simulated under an expansion. d) Demographic history simulated under a decrease. In addition we simulated data under four different germination rate *β*. β* equal the estimated germination rate.(TIF)

S14 FigEstimated demographic history in four simple demographic scenarios with seed banking where *μ* = 2.5 × 10^−9^.Estimated demographic history using four simulated sequences of 10 Mb under four different demographic scenarios with 10 replicates. Mutation and recombination rate are set to 2.5 × 10^−9^ per generation per bp. Simulation were done under four different germination rate b. We have *β* = 1 (red), 0.5 (blue), 0.2 (green) and 0.1 (purple). Therefore $\frac{r}{\mu }=1$ and respectively $\frac{\rho }{\theta }=1$, $\frac{\rho }{\theta }=0.5$, $\frac{\rho }{\theta }=0.2$ and $\frac{\rho }{\theta }=0.1$. The simulated demographic history is represented in black. a) Demographic history simulated under a constant population size. b) Demographic history simulated under a bottleneck. c) Demographic history simulated under an expansion. d) Demographic history simulated under a decrease. In addition we simulated data under four different germination rate *β*. β* equal the estimated germination rate.(TIF)

S15 FigEstimated demographic history with selfing under $\frac{r}{\mu }=5$.Estimated demographic history using four simulated sequences of 10 Mb and ten replicates under a saw-tooth demographic scenario (black). Simulation were done under four different self-fertilization rate *σ* (0,0.5,0.8 and 0.9). The mutation is set to 1.25 × 10^−8^ and the recombination rate to 6.25 × 10^−8^ per generation per bp. Therefore $\frac{r}{\mu }=5$ and respectively $\frac{\rho }{\theta }=5$, $\frac{\rho }{\theta }=3.335$, $\frac{\rho }{\theta }=1.667$ and $\frac{\rho }{\theta }=0.91$. Estimated demographic history are represented for all tested self-fertilization, *σ* = 1 (red), 0.5 (blue), 0.2 (green) and 0.1 (purple). The demographic history is estimated using a) eSMC where σ* equals the estimated self-fertilization rate, b) MSMC, c) MSMC2 and d) PSMC’.(TIF)

S16 FigEstimated demographic history in four simple demographic scenarios with selfing.Estimated demographic history using four simulated sequences of 10 Mb under four different demographic scenarios with 10 replicates. Mutation and recombination rate are set to 1.25 × 10^−8^ per generation per bp. Simulation were done under four different self-fertilization rate *σ* (0,0.5,0.8 and 0.9). Therefore $\frac{r}{\mu }=1$ and respectively $\frac{\rho }{\theta }=1$, $\frac{\rho }{\theta }=0.667$, $\frac{\rho }{\theta }=0.333$ and $\frac{\rho }{\theta }=0.182$. The simulated demographic history is represented in black. a) Demographic history simulated under a constant population size. b) Demographic history simulated under a bottleneck. c) Demographic history simulated under an expansion. d) Demographic history simulated under a decrease. In addition we simulated data under four different self-fertilization rate *σ*. We have *σ* = 0 (red), 0.5 (blue), 0.8 (green) and 0.9 (purple). σ* equal the estimated self-fertilization rate.(TIF)

S17 FigPossible selfing and seed banking value where $\frac{r}{\mu }=1$.Possible estimated self-fertilization and germination rates because of confounding effect using four simulated sequences of 10 Mb under a saw-tooth demographic scenario and four different combinations of germination (b) and self-fertilization (s) rate but resulting in the same $\frac{\rho }{\theta }=0.15$. Mutation rate is set to 1.25 × 10^−8^ and recombination rate to 1.25 × 10^−8^ per generation per bp. Therefore $\frac{r}{\mu }=1$. The four combination are: a) *σ* = 0.4 and *β* = 0.2, b) *σ* = 0.857 and *β* = 0.6, c) *σ* = 0.919 and *β* = 1 and d) *σ* = 0 and *β* = 0.15. Hence, for each scenario $\frac{\rho }{\theta }=0.15$. For each combination of *β* and *σ*, eSMC was launched with five different prior settings: ignoring seed banks and self-fertilization (red), accounting for seed banks and self-fertilization but without setting priors (blue), accounting for seed banks and self-fertilization with a prior set only for the self-fertilization rate (green), only for the germination rate (orange) or for both (purple).(TIF)

S18 FigEstimated demographic history with selfing and seed banking where $\frac{r}{\mu }=6.667$.Demographic history estimated by eSMC for ten replicates using four simulated sequences of 10 Mb under a saw-tooth demographic scenario and four different combinations of germination (b) and self-fertilization (s) rate but resulting in the same $\frac{\rho }{\theta }=1$. Mutation rate is set to 1.25 × 10^−8^ and recombination rate to 8.335 × 10^−8^ per generation per bp. Therefore $\frac{r}{\mu }=6.67$. The four combination are: a) *σ* = 0.4 and *β* = 0.25, b) *σ* = 0.75 and *β* = 0.6, c) *σ* = 0.85 and *β* = 1 and d) *σ* = 0 and *β* = 0.15. Hence, for each scenario $\frac{\rho }{\theta }=1$. For each combination of *β* and *σ*, eSMC was launched with five different prior settings: ignoring seed banks and self-fertilization (red), accounting for seed banks and self-fertilization but without setting priors (blue), accounting for seed banks and self-fertilization with a prior set only for the self-fertilization rate (green), only for the germination rate (orange) or for both (purple). σ* and β* respectively represent the estimated self-fertilization and germination rate.(TIF)

S19 FigPossible selfing and seed banking value where $\frac{r}{\mu }=6.667$.Possible estimated self-fertilization and germination rates because of confounding effect using four simulated sequences of 10 Mb under a saw-tooth demographic scenario and four different combinations of germination (b) and self-fertilization (s) rate but resulting in the same $\frac{\rho }{\theta }=1$. Mutation rate is set to 1.25 × 10^−8^ and recombination rate to 8.335 × 10^−7^ per generation per bp. Therefore $\frac{r}{\mu }=6.667$. The four combination are: a) *σ* = 0.4 and *β* = 0.2, b) *σ* = 0.857 and *β* = 0.6, c) *σ* = 0.919 and *β* = 1 and d) *σ* = 0 and *β* = 0.15. Hence, for each scenario $\frac{\rho }{\theta }=1$ For each combination of *β* and *σ*, eSMC was launched with five different prior settings: ignoring seed banks and self-fertilization (red), accounting for seed banks and self-fertilization but without setting priors (blue), accounting for seed banks and self-fertilization with a prior set only for the self-fertilization rate (green), only for the germination rate (orange) or for both (purple).(TIF)

S20 Fig Estimated demographic history of *Arabidopsis thaliana* where selfing and seed banking is ignored. Demographic history of two European (Sweden (blue) and German (green)) populations of *A*. *thaliana*. Mutation rate is set to 7 × 10^−9^ per generation per bp and was use as prior for recombination rate. a) Demographic history estimated by eSMC without accounting self-fertilzation or dormancy. b) Demographic history estimated by MSMC. c) Demographic history estimated by MSMC2. d) Demographic history estimated by PSMC’.
(TIF)
