# Supplementary material for: Inference of past demography, dormancy and self-fertilization rates from whole genome sequence data
Source: PLoS Genet. 2020 Apr 6;16(4):e1008698. doi: 10.1371/journal.pgen.1008698 (PMC7173940; doi:10.1371/journal.pgen.1008698)
Supplement: Attachment — Submitted filename: review_r.pdf [file pgen.1008698.s024.pdf]

Referee Report

The authors have produced a modified version of PSMC (called eSMC) which is designed to estimate population size history while taking into account certain non-standard features (selfing and dormancy) found in nature.

I found the paper stimulating in places, and there is definitely scope for extending the PSMC family of methods beyond the realm of human genetics to study other organisms with complex life-cycles. The authors compared their implementation with MSMC and MSMC2 on several simulated data sets. Not surprisingly, when data are simulated under their assumed model, their method does better. The authors even claim that eSMC also does a little bit better on data simulated under the standard coalescent, though the evidence (Figure 1) is fairly limited. It would be interesting to know more about how eSMC differs algorithmically from the existing implementations, or what the source of improvement is.

Unfortunately, the positive aspects of this manuscript are vitiated by some technical oversights which left me with an overall negative opinion.

First, I cannot see why a new method is even necessary here, or indeed whether eSMC is actually different from PSMC. It seems to me that the former is simply a trivial rescaling of the latter. Starting from section 5.1, let $c_1 = 2\beta^2/(2-\sigma)$ and $c_2 = \frac{c_1(2-\sigma)}{2\beta(1-\sigma)} = \beta/(1-\sigma)$. Then setting

$$\mu' = \mu/c_1 \tag{1}$$
$$r' = r/c_2 \tag{2}$$
$$T'_\alpha = c_1 T_\alpha = -\log(1-\alpha/k) \tag{3}$$
$$\Delta'_\alpha = T'_{\alpha+1} - T'_\alpha \tag{4}$$

we can rewrite equations (2)–(5) in section 5.1 as

$$\mathbb{P}(0 \mid \alpha) = 1 - e^{-2\mu' t'_\alpha} \tag{5}$$
$$\mathbb{P}(1 \mid \alpha) = 1 - \mathbb{P}(0 \mid \alpha) \tag{6}$$
$$t'_\alpha = \frac{T'_\alpha - T'_{\alpha+1} e^{-\Delta'_\alpha/\chi_\alpha}}{1 - e^{-\Delta'_\alpha/\chi_\alpha}} + \chi_\alpha \tag{7}$$
$$P'_\gamma = 1 - e^{-2r' t'_\gamma} \tag{8}$$
$$p(\alpha \mid \gamma) = \frac{P'_\gamma}{2t'_\gamma} \sum_{\eta=1}^{\alpha-1} \frac{(1-e^{-2/\chi_\alpha})}{2/\chi_\alpha} \left[e^{-2\int_{T'_{\eta+1}}^{T'_\alpha} \frac{d\nu}{\chi_\nu}} (1-e^{-2/\chi_\nu}) - 1 \right] + \Delta'_\alpha, \quad \alpha < \gamma, \tag{9}$$

et cetera.

If we denominate time in units of the mutation rate (i.e. setting $t''_\alpha := \mu'_\alpha t'_\alpha$) then the model given by the above equations is *exactly* PSMC'. Thus, estimates of $\hat{\beta}$ or $\hat{\sigma}$ obtained using eSMC are seemingly identical to what you would get

by estimating $\widehat{(r'/\mu')}$ using PSMC and then solving equations (1) and (2) for $c_1/c_2 = \beta/(1-\sigma)$, assuming as always that the biological rates of mutation and recombination are known.

(Note that the rescaling chosen by the authors leads to $\rho/\theta = \beta/(1-\sigma)(r/\mu)$, not $\beta(1-\sigma)(r/\mu)$ as stated in line 167; see comment below.)

Did the authors try this approach first? The discussion (specifically, lines 200-202 and 408-410) suggests that they are aware of it. I don't understand why the authors felt compelled to create an entirely new method, when seemingly all of the estimates in this paper could just as easily have been acquired using an existing program like MSMC. They should do more to justify why a new method is necessary.

Second, there is an identifiability issue here which is not properly acknowledged. As the authors note in section 2.1, under a standard coalescent-based model, the combined effects of parthenogesis and seed banking are to alter the ratio of recombination to mutation by a multiplicative function of β and σ. As argued above, we can estimate the value of that function using PSMC by dividing the estimated ratio $\widehat{\left(\frac{\rho}{\theta}\right)}$ by r/μ, the true biological recombination and mutation rates. This provides an estimator of *either* β or σ, *assuming that the other is known.* But β and σ *are* not jointly identifiable, and this remains true even if you add additional assumptions like $\beta \leq 1/2$ or $\sigma \geq 1/2$: just take any pair of parameters in this range for which $\beta(1-\sigma)$ is constant (for example, $\beta = 1/4, \sigma = 3/4$ and $\beta = 1/3, \sigma = 13/16$.) In light of this, it is not surprising that the authors observed in section 3.1.4 an upward bias when trying to jointly estimate β and σ: increasing both parameters by appropriate amounts produces the exact same data distribution! Without additional identifying assumptions or sources of data, they can't be disentangled.

The reason why I find this troubling is that this paper is obviously aimed at practitioners, who do not necessarily appreciate the notion of parameter identifiability. I can quite easily see this method being used to jointly estimate both parameters at once; in fact that may even be the default. Yet those estimates would be totally spurious and misleading.

Minor comments

- The authors state in line 167 that the ratio of effective recombination to mutation under their model is $\rho/\theta = [(1-\sigma)\beta](r/\mu)$. In line 186 they rescale time by a factor of $c_1 = (2-\sigma)/(2\beta^2)$ and in line 190 the recombination rate is scaled by $c_2 = 2(1-\sigma)\beta/(2-\sigma)$. So the number of recombinations per unit time is

  $$\rho = r/(c_1 c_2) = r\beta/(1-\sigma).$$

  Thus

  $$\rho/\theta = [\beta/(1-\sigma)](r/\mu)$$

which is different. Why the discrepancy?

- 501: “phased nucleotides”: PSMC does not require phased data, and the phrase itself does not make sense.
- Section 3.1: As this section does not contain any theoretical (mathematical) results, I suggest changing its title to “Simulation Results”.
- 393-395: doesn’t MSMC2 already use information from all pairs of samples?
- Minor grammatical or spelling errors on lines 130, 317, 425.
