# Supplementary material for: Inference of past demography, dormancy and self-fertilization rates from whole genome sequence data
Source: PLoS Genet. 2020 Apr 6;16(4):e1008698. doi: 10.1371/journal.pgen.1008698 (PMC7173940; doi:10.1371/journal.pgen.1008698)
Supplement: Attachment — Submitted filename: Response to reviewers.odt [file pgen.1008698.s025.odt]

­Detailed response to comments:

Dear Dr Sellinger,
Thank you very much for submitting your Research Article entitled 'Inference of past demography, dormancy and self-fertilization rates from whole genome sequence data' to PLOS Genetics. Your manuscript was fully evaluated at the editorial level and by 3 independent peer reviewers. The reviewers appreciated the attention to an important problem, but raised some substantial concerns about the current manuscript. The main concern expressed by several reviewers focuses on the novelty of this approach and whether it represents a real improvement over the approaches it is clearly based upon. We would emphasize that the authors pay close attention to addressing this question, along with others raised in the reviews.
Based on the reviews, however, we will not be able to accept this version of the manuscript, but we would be willing to review again a much-revised version. We cannot, of course, promise publication at that time. Should you decide to revise the manuscript for further consideration here, your revisions should address the specific points made by each reviewer. We will also require a detailed list of your responses to the review comments and a description of the changes you have made in the manuscript. If you decide to revise the manuscript for further consideration at PLOS Genetics, please aim to resubmit within the next 60 days.
If present, accompanying reviewer attachments are included with this email; please notify the journal office if any appear to be missing. They will also be available for download from the link below. You can use this link to log into the system when you are ready to submit a revised version, having first consulted our Submission Checklist<http://journals.plos.org/plosgenetics/s/submit-now#loc-submission-checklist.
 To enhance the reproducibility of your results, we recommend that you deposit your laboratory protocols in protocols.io, where a protocol can be assigned its own identifier (DOI) such that it can be cited independently in the future. For instructions see our guidelines <http://journals.plos.org/plosgenetics/s/submission-guidelines#loc-materials-and-methods.
 > We have included the pipeline to reproduce our multihetsep files from Daphnia pulex in the following github repository :  https://github.com/TPPSellinger/Daphnia_pulex_data

 Please be aware that our data availability policy<http://journals.plos.org/plosgenetics/s/data-availability requires that all numerical data underlying graphs or summary statistics are included with the submission, and you will need to provide this upon resubmission if not already present. In addition, we do not permit the inclusion of phrases such as "data not shown" or "unpublished results" in manuscripts. All points should be backed up by data provided with the submission.
 > The corresponding Figures (S9 and S10) have now been added.

 While revising your submission, please upload your figure files to the Preflight Analysis and Conversion Engine<http://pace.apexcovantage.com/ (PACE) digital diagnostic tool.  PACE helps ensure that figures meet PLOS requirements. To use PACE, you must first register as a user. Then, login and navigate to the UPLOAD tab, where you will find detailed instructions on how to use the tool. If you encounter any issues or have any questions when using PACE, please email us at figures@plos.org<mailto:figures@plos.org.
 > All figures have been processed through PACE.


We are sorry that we cannot be more positive about your manuscript at this stage. Please do not hesitate to contact us if you have any concerns or questions.
 Yours sincerely,
Rodney Mauricio, Ph.D.,  Associate Editor,  PLOS Genetics
Gregory P. Copenhaver, Editor-in-Chief,  PLOS Genetics
 
 
 ************************************
Reply to reviewers’ comments
Reviewer #1: The inference of historical population size changes from genome sequence data has received a lot of attention in recent years. Sellinger et al. build on previous work in the Tellier group by incorporating self-fertilization and seed dormancy into the established HMM-framework. They show that their method, which they call eSMC, infers population sizes in the presence of these confounding factors more reliably than the three published methods MSMC, MSMC2, and PSMC. The authors also find that dormancy has a stronger effect on estimation than selfing.
The authors supply two accompanying programs: scrm for simulating data under their model, and the R-package eSMC for estimation. The programs run as described, though the test script hasn't finished yet after over one hour and is producing a host of "unsuccessful convergence" messages. It would therefore much improve the paper if the authors supplied more extensive user documentation than their current README.
> We thank the reviewer for their comments and for the feed-back on the user-friendliness of our scripts. We have updated the current README to provide more detailed explanations. 
For a quick test of our method, we have now added three scripts for a complete tutorial (two of which do not require any skill or specific operating system). The tutorials can be completed in one hour and present a basic set of analyses.
The former computation time experienced by the reviewer was due to the high number of repetitions. With an average of 15-20 min per repetition, 10 repetitions lead to 3h of calculation time. The ‘unsuccessful convergence’ output is due to “BB”, an external package used for optimizing the objective function in a maximum number of iterations. Upon reaching this number, this error message is sent, but is of no consequence. We were unfortunately unable to mute such messages from the optimization function, and have now explained this in the README.

 Apart from that, eSMC looks like a useful addition to the popgen Toolkit. My suggestions for further improving the paper concern again mainly the computational side:
 
 - p. 9, l 207ff : "our implementation in R is thus slightly slower, but still allows whole genome sequence analysis of several individuals in reasonable amount of time". This could be made more concrete: How
 does the run time of eSMC vary as a function of the number of polymorphisms, individuals, etc.?
 - p. 17, l. 393f : The authors explain that the greater accuracy of their model is due to running all pairwise analyses. This sounds like a classical trade-off between accuracy and computational effort. Again, a systematic study of run time as a function of pertinent parameters would be helpful to users wishing to apply eSMC.
 > In reply to both comments, we have now added table 3 which gives computation times on a Desktop computer for sequence lengths of 1,10 and 30 Mb and three different numbers of haplotypes (2,4 and 10).

 - p. 45, Table 2: This contains only the averages of the results returned by the various methods. Variances would also be useful for establishing their relative merits.
 > We have now added the coefficient of variation (standard deviation over the average) to table 2. We chose the coefficient of variation rather than variance as the variance is linked to the average estimated value.

 - Minor: The authors repeatedly refer to "coalescent times" (e.g. p. 7, l. 151) where they might prefer to write "coalescence times" instead.
 > Corrected.


Reviewer #2 : The paper presents a new method to infer demographic history and two important ecological parameters from genomic data, namely the self-fertilization rate and seed dormancy. The model is essentially a modified PSMC/PSMC' model, and the two additional parameters are important within plant population genetics and ecology. I think the key model and results are supported by evidence, and the authors have already provided many tests and simulations to assess the accuracy of their method and to compare with previous methods.
Before I outline some comments, as a general note : Providing the figures completely separate from the captions (and not even in the right order), and again completely separate from the main text makes life unnecessarily hard for reviewers! I think PLOS Genetics should encourage authors to submit nicely integrated documents to be sent to the reviewers instead of this puzzle work where one has to go back and forth between main text, figure captions and actual figures.
> We apologize for having not included the figures with the caption, making it more difficult in an already difficult-to-read layout. We have attempted to make our submission file as reader/reviewer friendly as possible, while still following PloS guidelines.
 
 I have some major requests for clarification and comments:
 - I generally don't fully understand how this model (eSMC) is different from MSMC2 or PSMC'. As far as I understand from the model description (section 2 in the main text) and SI 1, eSMC is _exactly_ a PSMC' model, but with modified coalescence and recombination rates. If that is the case, then MSMC2 with a freely inferred recombination rate, and freely inferred coalescence rates, should be exactly equivalent to eSMC after rescaling those coalescence and recombination rates, or do I miss something? I think it's totally fine to reimplement it and integrate the two additional parameters beta and sigma more tightly into the inference framework, but the reader should be fully aware of what the differences to previous methods are. If the only difference are i) a re-interpretation of the effective coalescence and recombination rates obtained by PSMC', and ii) the pairwise running-scheme over all haplotypes (which is already the case in MSMC2 I think), then that should be said more clearly.
> This concern is shared with reviewer 3 and we have now clearly stated that our theoretical model is a rescaled PSMC' (page 8, line 180). Note that for MSMC2, the method description is not yet published in a journal (on the 17/10/2019), but available on bioaRxiv doi: https://doi.org/10.1101/585265. We therefore contacted the author, Dr Stefan Schiffels, in order to provide answers to some of the reviewers' questions. In brief, in addition to explaining that our implementation is an extention to PSMC', we have also included a list of the differences between our method and existing ones (now on page 10, lines 214-229 of the manuscript):
1) We re-interpret the effective coalescence and recombination rates obtained by PSMC', offering new biological interpretations of the parameters. 
2) We implement a pairwise analysis scheme running over all haplotypes.
3) We include the possibility to optimize the likelihood under constraint. This means that the user can bound parameter values in order to integrate existing knowledge and avoid any estimations that would make no biological or ecological sense (particularly suited for ecologists with prior knowledge).
4) In our implementation we change the emission matrix to avoid program crashes. PSMC’ and MSMC are designed for human data, hence the linearization of the mutation probability makes sense. However, for other species with extremely strong seed banks, high effective population size or very high mutation rates, this linearization is not only no longer appropriate, but can lead to negative emission probabilities (resulting in crashing the algorithm). Note that MSMC2 does not linearize and does not result in this kind of crash (S. Schiffels personal communication).
5) eSMC differs from MSMC (and MSMC2) in its implementation of the Baum-Welch algorithm. We compute the value of the objective function at every position, but in MSMC (and MSMC2), in order to speed up the algorithm, it is only calculated every 1000 bp (S. Schiffels personal communication and supplementary material page 9 of doi: https://doi.org/10.1101/585265). This explains why on the one hand, eSMC is slower, but on the other,  our method better extracts information from small datasets.
6) The objective function used in eSMC is similar to that used in MSMC and PSMC‘, however MSMC2 uses a different function ( Supplementary Material of http://dx.doi.org/10.1101/585265 ).
7) PSMC’, MSMC and MSMC2, are designed for human data where long scaffolds (>10 Mb) are available. Our method is designed to extract the maximum amount of information from small datasets, as is often the case in non-model species (e.g. Daphnia pulex where the longest scaffold < 2Mb).

- Given the above point, I don't understand why the results between eSMC and MSMC2 are so different in terms of variance of results. Why has eSMC so much less variance in recent times compared to MSMC2? Specifically the differences between Figure S3 (eSMC) and S4 (PSMC) are striking. I can think of no good reason why eSMC should have less variance than MSMC2. It runs on the same amount of data and has the same underlying model. Could this be a bug in MSMC2?
 > We have now included the reasons why other methods have higher variance in the Discussion (line 446-452). Concerning the observed differences with MSMC2: MSMC2 has a wider window than eSMC, and thus estimates more recent and also older events. MSMC2 actually uses the same time window as the original PSMC (Li & Durbin 2011), hence attempting to estimate more information from the same amount of data used in other methods. In addition, MSMC2 does not use the same objective function (or composite likelihood) as MSMC, PSMC‘ and eSMC to estimate parameters. As a result, MSMC2 estimates parameters on a different time window and with a different objective function, the estimated parameters therefore differ from other methods.

- I don't understand whether there is _any_ information that allows eSMC to separately and simultaneously estimate sigma and beta. The authors are a bit murky on that. In lines 167ff they say "We therefore have a potential confounding effect between self-fertilization and seed-banking when observing the recombination and mutation ratio". Here the word "potential" should be removed, as it looks to me mathematically impossible to infer them both simultaneously, unless I misunderstand something. And in line 171ff "seed-banking can theoretically be equivalent to self-fertilization with higher effective population size". If I understand correctly, it is not just "theoretically equivalent" but "fully equivalent", and it shouldn't sound as if this was just a mere possibility or "potential problem".
 > We thank the reviewer for pointing this out, as it was never our intention to hide the issue of non-identifiability between the estimations of sigma and beta . We elaborate on this point below in reply to reviewer 3, and have now more explicitly stated  that it is not possible to distinguish seed banks from self-fertilization (page 19, line 439).

The discussion in section 3.1.4 doesn't make it any clearer. For example, in Lines 323ff it says: "Without any prior knowledge it is not possible for eSMC to estimate to correct set of parameters." And further below, that estimates "can be slightly biased"... I don't understand. IS there, or ISN'T there information in the data that allows in principle to disentangle the two? If there isn't, then I would expect the results not to be "slightly biased", but completely uniformly distributed within the priors.
> We now explicitly state that estimating simultaneously both will lead to biases (page 16 line 365). Following suggestion by reviewer 3, we have added a warning on the github when users attempt to estimate both parameters. We have also added a new function to plot possible values of beta and sigma to help users, as well as figures produced when using this function, which can be found in the SI ( S17 and S19). However,  although there is an identifiability problem, prior knowledge limits this and our method does detect strong effects of sigma or beta (see lines 439-443 in Discussion)

 - Simulations: MSMC, MSMC2 and PSMC default parameters are designed for about 100 times as much data. With this little data as used here (1, 10 and 30 Mb), arguably the time discretisation should be adapted. For example, I would suggest that for PSMC and MSMC2 to use the exact same patterning as in eSMC to make it more comparable. With MSMC this is difficult, since the distribution of first coalescence times has a different window than the the distribution of pairwise coalescence times (as used in eSMC, MSMC2 and PSMC), but at least for PSMC and MSMC2 this should easily be possible. And for MSMC at least the total number of time segments should be made equal to the number of segments used in eSMC.
 > We thank the reviewer for pointing this out. We have now added a supplementary figure (Figure S2), where all methods have the same discretisation of population size  and patterning. However, as stated by the reviewer, by definition MSMC (with a number of haplotypes greater than two) and MSMC2 have different time windows than eSMC or PSMC' (even after discussion with S. Schiffels). From Figure S2 we see that PSMC',and MSMC do indeed exhibit smaller variances when adapting the discretization to the size of the dataset, however eSMC still exhibits the smallest variance in the estimates. (Cannot change time windows and we did not)
 
Minor comments:
 - The main figures are all pretty poorly styled. There is a _lot_ of white space around the actual curves, which are squished into relatively tight log-spaces. I would recommend to zoom in a bit to focus on the actual estimates and curves.
 > All the figures have been changed so as to minimize the white spaces around the estimated demography.

 - What exactly is meant by PSMC'? Specifically, what software was used? PSMC, MSMC2 (with two phased haplotypes) or MSMC (with two phased haplotypes)? Command lines should be listed somewhere, sorry if I overlooked them.
 >  PSMC' is defined as MSMC using only 2 haplotypes This definition has been added in line 123 . The command lines are listed in the Supplementary file 2.

 - Table 2: Given the centrality of the rho/mu estimates in this paper, I think a simple average is not enough here. I would prefer some scatter plots, or Violin/Boxplots type charts to indicate what the variability of estimates with each of the methods is.
 > As mentioned above in our reply to reviewer 1, who had raised similar concerns, we have now included the coefficient of variation in table 2 . 

 - L 261ff and other places: I was unclear on whether the rho/mu ratio is generally kept free to be inferred, or whether it's fixed.
 > This has now been clarified throughout the Results. We now explicitly state for each analysis whether rho/theta is free to be inferred or not. 

 - Fig 2a: Is there a bug in the legend? The red curve should have beta=1, not 0, right? Same thing in Figure S9a I think.
 > Thank you for pointing this out. It was a typo that has now been corrected. 

 - Fig S15 typo "Sweeden".
 > Corrected

 - Figure 6 has no discussion on why the curves look like they look. It is simply said "The inferred demographic history of a single population of D. pulex is qualitatively similar using eSMC and PSMC (Fig 6)". But the curves are shifted a lot (vertically). Please add at least a sentence to discuss this, otherwise the uninitiated reader will have trouble aligning that graphic with the statement of being "qualitatively similar".
> We have now included a more detailed explanation to this upward shift, which demonstrates exactly why our method can be more appropriate for a biological understanding of the results compared to PSMC’ (line 404-406).


Reviewer 3 :
The authors have produced a modified version of PSMC (called eSMC) which is designed to estimate population size history while taking into account certain non-standard features (selﬁng and dormancy) found in nature. I found the paper stimulating in places, and there is definitely scope for extending the PSMC family of methods beyond the realm of human genetics to study other organisms with complex life-cycles. The authors compared their implementation with MSMC and MSMC2 on several simulated data sets. Not surprisingly, when data are simulated under their assumed model, their method does better. The authors even claim that eSMC also does a little bit better on data simulated under the standard coalescent, though the evidence (Figure 1) is fairly limited. It would be interesting to know more about how eSMC diﬀers algorithmically from the existing implementations, or what the source of improvement is. 
> We thank the reviewer for these positive comments. As reviewer 2 has made a similar comment, please see above for a description of the differences between the methods. This is now also included in the “Overview of the model” section (page 10, lines 214-229) .

Unfortunately, the positive aspects of this manuscript are vitiated by some technical oversights which left me with an overall negative opinion. First, I cannot see why a new method is even necessary here, or indeed whether eSMC is actually diﬀerent from PSMC. It seems to me that the former is simply a trivial rescaling of the latter.
> From the mathematical point of view (on expectation), the reviewer is correct, and we now explicitly state that our method is a rescaled PSMC’ (page 8, line 180). As mentioned by reviewer 2 , PSMC’, MSMC and MSMC2, are designed for human data, where long scaffolds (>10 Mb) are available. Our method is designed to extract the maximum amount of information from small datasets (e.g. Daphnia pulex where the longest scaffold < 2Mb). For short sequences, PSMC’ and MSMC display a higher variance. MSMC2 is oriented towards predicting demographic history and population structure, but poorly estimates recombination rates, which are crucial for our work. It is therefore necessary to provide methods designed for small datasets and, more generally, for species with  small genomes.

Did the authors try this approach ﬁrst? The discussion (speciﬁcally, lines200-202 and 408-410) suggests that they are aware of it. I don’t understand why the authors felt compelled to create an entirely new method, when seemingly all of the estimates in this paper could just as easily have been acquired using an existing program like MSMC. They should do more to justify why a new method is necessary.
> Indeed, as the reviewer suggests, it is possible to rescale the output of MSMC or PSMC’ (but not MSMC2 because it estimates the recombination rate too poorly for any pertinent post-processing), and we had initially attempted this approach. However, we found that the resulting behavior was unsatisfactory for small datasets (as for D. pulex) and when considering seed banks. In species with seed banks, the high observed diversity can result in negative probability, and thus it was necessary to build a method with a corrected emission matrix to avoid this outcome. Furthermore, our method estimates simultaneously the past demography and the rate of seed bank using ecological priors, thus providing a biological explanation to shifts of demographic estimations (in population size and in time) that could be overlooked if rescaling were to take place a postiriori. We have detailed these improvements more explicitly in the Introduction and Materials and Methods.

Second, there is an identiﬁability issue here which is not properly acknowledged. As the authors note in section 2.1, under a standard coalescent-based model, the combined eﬀects of parthenogesis and seed banking are to alter the ratio of recombination to mutation by a multiplicative function of β and σ. As argued above, we can estimate the value of that function using PSMC by dividing the estimatedratiod ρ θby r/µ, thetruebiologicalrecombinationandmutationrates.This provides an estimator of either β or σ, assuming that the other is known. But β and σ are not jointly identiﬁable, and this remains true even if you add additional assumptions like β ≤1/2 or σ ≥1/2: just take any pair of parameters in this range for which β(1−σ) is constant (for example, β = 1/4,σ = 3/4 and β = 1/3,σ = 13/16.) In light of this, it is not surprising that the authors observed in section 3.1.4 an upward bias when trying to jointly estimate β and σ: increasing both parameters by appropriate amounts produces the exact same data distribution! Without additional identifying assumptions or sources of data, they can’t be disentangled. The reason why I ﬁnd this troubling is that this paper is obviously aimed at practitioners, who do not necessarily appreciate the notion of parameter identiﬁability. I can quite easily see this method being used to jointly estimate both parameters at once; in fact that may even be the default. Yet those estimates would be totally spurious and misleading. 
> We apologize for the lack of clarity, as it was never in our intention to hide the non-identifiability problem between estimations of sigma and beta. This has now been clarified (page 19, line 439) and we have included explicit guidelines for users when simultaneously estimating these two variables. We also provide a new function (available on github) which allows the user to view the possible combinations of selfing and germination rates which could explain the data, as well as two figures in the Supplementary Material (S17 Fig and S19 Fig). However,  it is important to note that prior knowledge can reduce the scope of possible values (and large effects of selfing or seed bank can be recovered within a given range of ecological priors).

Concerning the discrepancy of the ratio of recombination over mutation (computations in the file attached): 
>We are very grateful to reviewer 3 for having taken the time to recalculate the rates and  pointing out the error in our computations. We had indeed miscalculated the ratio rho/theta in presence of self-fertilization (see now equation 2 for the short demonstration and corrected formula). However, there was a term missing in the reviewer's calculations, and the actual correct ratio is slightly different than that obtained by the reviewer. The new results and their demonstration can be found in equation 2. Please note that this had no effect on the estimations, as this ratio was only taken into account during the search for priors. We have nonetheless relaunched all of the simulations in which it had been involved, and as expected, found no significant differences in the results (Figure 3 ,Figure 4,Figure 5, S15 Fig, S16 Fig  and S18 Fig were redone for completeness with the new prior formula).


501: “phased nucleotides”: PSMC does not require phased data, and the phrase itself does not make sense.
> This has been changed (line 553)

Section 3.1: As this section does not contain any theoretical (mathematical) results, I suggest changing its title to “Simulation Results”.
> Corrected.

393-395: doesn’t MSMC2 already use information from all pairs of samples?
> Yes, the reviewer is correct, and we have included this information in the discussion on lines 447.

Minor grammatical or spelling errors on lines 130, 317, 425
> Corrected.
