# Supplementary material for: Inference of past demography, dormancy and self-fertilization rates from whole genome sequence data
Source: PLoS Genet. 2020 Apr 6;16(4):e1008698. doi: 10.1371/journal.pgen.1008698 (PMC7173940; doi:10.1371/journal.pgen.1008698)
Supplement: Attachment — Submitted filename: Response to reviewers.odt [file pgen.1008698.s026.odt]

­Detailed response to comments:

Dear Dr Sellinger,

Thank you very much for submitting your Research Article entitled 'Inference of past demography, dormancy and self-fertilization rates from whole genome sequence data' to PLOS Genetics. Your manuscript was fully evaluated at the editorial level and by three independent peer reviewers. The reviewers appreciated the attention to an important topic but identified some aspects of the manuscript that should be improved. You should pay close attention to Reviewer 3's comments. Since two reviewers recommend tightening the Discussion, you should make  all efforts to shorten that section as well.
We therefore ask you to modify the manuscript according to the review recommendations before we can consider your manuscript for acceptance. Your revisions should address the specific points made by each reviewer.
In addition we ask that you:
1) Provide a detailed list of your responses to the review comments and a description of the changes you have made in the manuscript.
2) Upload a Striking Image with a corresponding caption to accompany your manuscript if one is available (either a new image or an existing one from within your manuscript). If this image is judged to be suitable, it may be featured on our website. Images should ideally be high resolution, eye-catching, single panel square images. For examples, please browse our archive. If your image is from someone other than yourself, please ensure that the artist has read and agreed to the terms and conditions of the Creative Commons Attribution License. Note: we cannot publish copyrighted images.

Please find in the uploaded documents an image (named striking_image.tiff) capturing the essence of our work.

We hope to receive your revised manuscript within the next 30 days. If you anticipate any delay in its return, we would ask you to let us know the expected resubmission date by email to plosgenetics@plos.org.
If present, accompanying reviewer attachments should be included with this email; please notify the journal office if any appear to be missing. They will also be available for download from the link below. You can use this link to log into the system when you are ready to submit a revised version, having first consulted our Submission Checklist.
While revising your submission, please upload your figure files to the Preflight Analysis and Conversion Engine (PACE) digital diagnostic tool. PACE helps ensure that figures meet PLOS requirements. To use PACE, you must first register as a user. Then, login and navigate to the UPLOAD tab, where you will find detailed instructions on how to use the tool. If you encounter any issues or have any questions when using PACE, please email us at figures@plos.org.
Please be aware that our data availability policy requires that all numerical data underlying graphs or summary statistics are included with the submission, and you will need to provide this upon resubmission if not already present. In addition, we do not permit the inclusion of phrases such as "data not shown" or "unpublished results" in manuscripts. All points should be backed up by data provided with the submission.
We added a zip file entitled “numerical_data.zip” containing all the files required for figures and summary statistics.

PLOS has incorporated Similarity Check, powered by iThenticate, into its journal-wide submission system in order to screen submitted content for originality before publication. Each PLOS journal undertakes screening on a proportion of submitted articles. You will be contacted if needed following the screening process.
To resubmit, you will need to go to the link below and 'Revise Submission' in the 'Submissions Needing Revision' folder.
Please let us know if you have any questions while making these revisions.
Yours sincerely,

Rodney Mauricio, Ph.D.
Associate Editor
PLOS Genetics

Gregory P. Copenhaver
Editor-in-Chief
PLOS Genetics


***********************************************
Reviewer's Responses to Questions
Comments to the Authors:
Please note here if the review is uploaded as an attachment.

Reviewer #1: All my comments have been addressed.

Reviewer #2: I think the manuscript has improved and most of my issues have been addressed.
Just some minor points:
1) L 258: should reference Table 2, not Table 3.
Corrected at line 258

2) Figure 3 is not referenced from the main text.
Corrected at line 343

3) L 366 "[...], as there is an identifiability issue in the case of joint inference". I would say "[...], since the separate rates are non-identifiable" or something clearer.
Corrected. The paragraph on lines 360-376 has been modified accordingly.

4) L 439 "In its current version, our model cannot disentangle the genomic signatures of
self-fertilization and seed-banks". This sounds like this was a technical or implementation issue, but it is fundamental, and so I would remove "In its current version".
Corrected. The paragraph on lines 440-443 has been modified accordingly.

5) The discussion is very long, and I think it contains some redundancy. The paragraph starting from line 463 overlaps with the one from line 515, so perhaps things can be shortened a bit.
The highlighted paragraph was shortened (lines 440-443), and we suppress the redundancy by removing  lines 509-513.

Reviewer #3: I thank the authors for their revisions. As best I can tell, the current state of the manuscript/this method is as follows:
1. eSMC is the same model as PSMC' (aka MSMC with sample size n=1 diploid; line 180).
2. The time discretization that eSMC uses is different from the defaults used by these methods (266ff). Note that it is possible to alter the default time discretization of PSMC/MSMC using the -p option, but the authors do not seem to have tried this.
3. Like MSMC2, eSMC uses a composite likelihood over all samples, as well as the correct Poisson likelihood for emission data, instead of a linear approximation which assumes that \theta * N_e << 1. (269, 562ff).
4. eSMC optimizes over additional selfing and dormancy parameters during the M-step, and also incorporates parameter constraints.
5. eSMC operates on raw sequence data, instead of aggregating observations in window sizes of 100 or 1000.
Features 1-3 are not new. Feature 4 is, as noted by multiple reviewers, not necessary at all; one of these parameters can be backed out from the estimate of \hat{\rho} and the other cannot be identified.
Reviewer  #3 summarized what we explained in our previous extensive revised version.

Feature 5 assumes that individual 100/1000bp blocks are non-recombining. I would not expect it to have a major impact on inference unless the recombination rate is exceptionally high, which is not the case here.
Though, when considering human polymorphism data, this statement is indeed correct, we slightly disagree with it for two fundamental reasons that highlight the novelty of our approach.
First, our study is oriented towards non-human data, and it can be expected that numerous species exhibit very high effective recombination rates violating the hypothesis of no-recombination within 100-1000bp blocks (see for example the recombination rates in insects such as Drosophila melanogaster or fungal crop pathogens such as Zymoseptori tritici). 
Second, and more importantly, the effective recombination is the result of both the per site  recombination rate and the time to the most recent common ancestor. For species with seed banks, one of the ecological traits we have focused on in this paper, small germination rates (strong seed banks) generate very large coalescence times, thus increasing the probability of recombination events. Hence, even in species with small per site recombination rates, the effective recombination will appear very large, violating the hypothesis of no recombination in 100/1000 bp blocks. This has been previously suggested (see for example Tellier New Phytologist 2019) and we have tested it theoretically, as can be seen in Figure 2 of our manuscript. Specifically, the violation of this hypothesis partially explains the observed bias in the estimation of the demographic history in the far past, especially obvious in the results obtained using MSMC2 than for the other models,  because the estimation goes further back in the past.

All of these could be achieved by either altering the default command-line settings MSMC, or making minor coding modifications. But I appreciate that it is not reasonable to expect practitioners to do this, so a new implementation can be valuable.
We thank reviewer #3 for acknowledging the value of our novel implementation and usefulness for empirical population geneticists.

Still, I think the most useful information contained in this paper is how to best parameterize {P,M}SMC for non-human data. In particular, setting the time discretization correctly can have a big impact on the quality of the estimates, as has been shown recently by other authors (Parag & Pybus, 2019). This seems to be the main source of improvement of their method over earlier methods -- it has a better default discretization for this particular type of data. In general, the authors have done a lot of simulation work exploring what works and does not work when running coalescent HMMs on non-human data. 
We again thank reviewer #3 for acknowledging the value of our work and our recommendations for the analysis of non-human data. In the discussion we have included the reference of the study conducted by  Parag & Pybus (line 518), offering extra guidelines for users wishing to use methods based on the sequentially markovian coalescent. 

Those findings are mostly relegated to the supplemental. To me this is the primary contribution of this work, and should expanded to become the main focus of the manuscript, rather than the novelty of eSMC per se.
Though we do understand the reviewer's point of view, we would prefer to keep the balance of results between main text and supplement files as it is for three reasons. First, the main aim of our study is to provide a novel approach to infer life history traits using whole genome sequence data. This obviously cannot be done in humans, and thus our implementation is optimized for non-human species and smaller datasets. Second, we believe that our figures in the main text provide evidence that our method is more appropriate for such cases, and thus we offer solutions for biologists analyzing non-human species with those ecological traits. Thirdly, as is usually the case, the supplement files are heavy and very technical. Moving these analyses into the main text would therefore drastically deteriorate the readability of our manuscript (especially for practitioners).

Finally, the discussion around identifiability remains unsatisfactory, and actually became less clear in revision as it is now a hodgepodge of the original (erroneous) assertions, and the reviewer comments which contradict them. A clear, unambiguous statement that the parameters cannot be jointly estimated is all that is needed here.
We are sorry that our modifications had not clarified this point. We have now modified the text on lines 360-376 and 440-448 following the answers to Reviewer #2 (see above), and hope that this is now stated clearly and unambiguously.

I did not find the claimed "explicit guidelines for users when simultaneously estimating these two variables" anywhere.
We apologize for this misunderstanding, we referred to the GitHub. The warnings are programmed to appear when launching the method (i.e. the Rcode) if the users attempt to infer both parameters. The explicit guidelines are of course also on our GitHub repository (written as warnings see : https://github.com/TPPSellinger/eSMC ).

In my opinion, the only guideline necessary is that this should not be done. The authors seem to want to write this off as an issue of bias, but it is much worse than that. Biased estimators can be consistent, but unidentifiable models can never be consistently estimated, even in the presence of priors.
We agree that our method should not be used to simultaneously infer selfing and dormancy based on the genome data only (without any kind of prior knowledge). This is now clearly stated in the manuscript (lines 360-376 and 440-443) and on the github. We changed the term bias on lines 360-376 and 440-443 to further avoid confusion.
Though with prior knowledge on the species ecological or life-history traits, the non-identifiability is still an issue, it becomes restricted to a much smaller area of the parameter space (thus resulting in small error rates for both parameters). Though the values will not be exact, we do believe that our method would still be of great help to empirical geneticists and ecologists to whom even estimations of these variables could be quite informative.
Indeed, ecologists might not require to know the exact selfing rate, but rather know a more or less large confidence interval, e.g. between 60 and 70%. Furthemore, ecologists might be interested in the significant presence of dormancy or selfing (both below or above a threshold of 0.5). We show that with good enough priors, our method can provide a first answer to the question: how significant are the consequences of these life history traits on polymorphism throughout the genome? Overall, we believe that, when used with proper caution, our method allows the incorporation of prior ecological incertitude by displaying all possible germination/selfing rates which can fit the data.
