# Supplementary material for: Correction: Inference of past demography, dormancy and self-fertilization rates from whole genome sequence data
Source: PLoS Genet. 2021 Apr 7;17(4):e1009504. doi: 10.1371/journal.pgen.1009504 (PMC8026070; doi:10.1371/journal.pgen.1009504)
Supplement: S2 Appendix — Contains all scrm command line to reproduce all tested scenario. (PDF) [file pgen.1009504.s001.pdf]

Appendix

Thibaut Sellinger[1], Diala Abu Awad[1], Markus Möst[2], Aurélien Tellier[1]

[1] Department of Population Genetics, Technische Universität München

[2] Department of Ecology, University of Innsbruck

January 15, 2021

1 Command lines for simulated data

Figure 1 :

./scrm 4 1 -t 15000 -r 15000 3e+07 -eG 5e-04 6437.7516497364 -eG 0.00075 -378.691273513906-eG 0.005 -643.77516497364 -eG 0.0075 37.8691273513906 -eG 0.05 64.377516497364 -eG 0.075 -3.78691273513906 -eG 0.5 -6.4377516497364 -eG 0.75 0.378691273513906 -eG 5 0.64377516497364 -eG 7.5 -0.0378691273513906 -eG 50 -0.064377516497364 -eG 75 0.00378691273513906 -eN 500 1 -B 1 1 -S 1 0 > fig2.txt

Supplementary Figure 1,2 :

./scrm 4 1 -t 5000 -r 5000 1e+07 -eG 5e-04 6437.7516497364 -eG 0.00075 -378.691273513906-eG 0.005 -643.77516497364 -eG 0.0075 37.8691273513906 -eG 0.05 64.377516497364 -eG 0.075 -3.78691273513906 -eG 0.5 -6.4377516497364 -eG 0.75 0.378691273513906 -eG 5 0.64377516497364 -eG 7.5 -0.0378691273513906 -eG 50 -0.064377516497364 -eG 75 0.00378691273513906 -eN 500 1 -B 1 1 -S 1 0 > figs1.txt

Supplementary Figure 3 :

./scrm 4 1 -t 500 -r 500 1e+06 -eG 5e-04 6437.7516497364 -eG 0.00075 -378.691273513906-eG 0.005 -643.77516497364 -eG 0.0075 37.8691273513906 -eG 0.05 64.377516497364 -eG 0.075 -3.78691273513906 -eG 0.5 -6.4377516497364 -eG 0.75 0.378691273513906 -eG 5 0.64377516497364 -eG 7.5 -0.0378691273513906 -eG 50 -0.064377516497364 -eG 75 0.00378691273513906 -eN 500 1 -B 1 1 -S 1 0 > figs3.txt

Supplementary Figure 4 a), 5 a), 6 a), 7 a):

./scrm 4 1 -t 5000 -r 5000 1e+07 -B 1 1 -S 1 0 > figs4a.txt

Supplementary Figure 4 b), 5 b), 6 b), 7 b):

./scrm 4 1 -t 5000 -r 5000 1e+07 -eN 0.3 0.1 -eN 2 1 -B 1 1 -S 1 0 > figs4b.txt

Supplementary Figure 4 c), 5 c), 6 c), 7 c):

./scrm 4 1 -t 5000 -r 5000 1e+07 -eG 0.05 3.576528694298 -eN 0.5 0.2 -B 1 1 -S 1 0 > figs4c.txt

Supplementary Figure 4 d), 5 d), 6 d), 7 d):

./scrm 4 1 -t 5000 -r 5000 1e+07 -eG 0.05 -3.576528694298 -eN 0.5 5 -B 1 1 -S 1 0 > figs4d.txt

Supplementary Figure 8,10,11 :

./scrm 4 1 -t 5000 -r 25000 1e+07 -eG 5e-04 6437.7516497364 -eG 0.00075 -378.691273513906-eG 0.005 -643.77516497364 -eG 0.0075 37.8691273513906 -eG 0.05 64.377516497364 -eG 0.075 -3.78691273513906 -eG 0.5 -6.4377516497364 -eG 0.75 0.378691273513906 -eG 5 0.64377516497364 -eG 7.5 -0.0378691273513906 -eG 50 -0.064377516497364 -eG 75 0.00378691273513906 -eN 500 1 -B 1 1 -S 1 0 > figs8.txt

Supplementary Figure 9 :

./scrm 4 1 -t 5000 -r 500000 1e+07 -eG 5e-04 6437.7516497364 -eG 0.00075 -378.691273513906-eG 0.005 -643.77516497364 -eG 0.0075 37.8691273513906 -eG 0.05 64.377516497364 -eG 0.075 -3.78691273513906 -eG 0.5 -6.4377516497364 -eG 0.75 0.378691273513906 -eG 5 0.64377516497364 -eG 7.5 -0.0378691273513906 -eG 50 -0.064377516497364 -eG 75 0.00378691273513906 -eN 500 1 -B 1 1 -S 1 0 > figs9.txt

Figure 2 :
for β in 1,0.5,0.2,0.1
./scrm 4 1 -t 5000 -r 5000 1e+07 -eG 5e-04 6437.7516497364 -eG 0.00075 -378.691273513906-eG 0.005 -643.77516497364 -eG 0.0075 37.8691273513906 -eG 0.05 64.377516497364 -eG 0.075 -3.78691273513906 -eG 0.5 -6.4377516497364 -eG 0.75 0.378691273513906 -eG 5 0.64377516497364 -eG 7.5 -0.0378691273513906 -eG 50 -0.064377516497364 -eG 75 0.00378691273513906 -eN 500 1 -B 1 β -S 1 0 > fig2.txt

Supplementary Figure 12 :

for β in 1,0.5,0.2,0.1

./scrm 4 1 -t 1000 -r 1000 1e+07 -eG 5e-04 6437.7516497364 -eG 0.00075 -378.691273513906-eG 0.005 -643.77516497364 -eG 0.0075 37.8691273513906 -eG 0.05 64.377516497364 -eG 0.075 -3.78691273513906 -eG 0.5 -6.4377516497364 -eG 0.75 0.378691273513906 -eG 5 0.64377516497364 -eG 7.5 -0.0378691273513906 -eG 50 -0.064377516497364 -eG 75 0.00378691273513906 -eN 500 1 -B 1 β -S 1 0 > figs12.txt

Supplementary Figure 13 :

for β in 1,0.5,0.2,0.1

a):
./scrm 4 1 -t 5000 -r 5000 1e+07 -B 1 β -S 1 0 > figs13a.txt

b):
./scrm 4 1 -t 5000 -r 5000 1e+07 -eN 0.3 0.1 -eN 2 1 -B 1 β -S 1 0 > figs13b.txt

c):
./scrm 4 1 -t 5000 -r 5000 1e+07 -eG 0.05 3.576528694298 -eN 0.5 0.2 -B 1 β -S 1 0 > figs13c.txt

d):
./scrm 4 1 -t 5000 -r 5000 1e+07 -eG 0.05 -3.576528694298 -eN 0.5 5 -B 1 β -S 1 0 > figs13d.txt

Supplementary Figure 14 :

for β in 1,0.5,0.2,0.1

a):
./scrm 4 1 -t 1000 -r 1000 1e+07 -B 1 β -S 1 0 > figs14a.txt

b):
./scrm 4 1 -t 1000 -r 1000 1e+07 -eN 0.3 0.1 -eN 2 1 -B 1 β -S 1 0 > figs14b.txt

c):
./scrm 4 1 -t 1000 -r 1000 1e+07 -eG 0.05 3.576528694298 -eN 0.5 0.2 -B 1 β -S 1 0 > figs14c.txt

d):
./scrm 4 1 -t 1000 -r 1000 1e+07 -eG 0.05 -3.576528694298 -eN 0.5 5 -B 1 β -S 1 0 > figs14d.txt

Figure 3 :

for σ in 0,0.5,0.8,0.9

./scrm 4 1 -t 5000 -r 5000 1e+07 -eG 5e-04 6437.7516497364 -eG 0.00075 -378.691273513906-eG 0.005 -643.77516497364 -eG 0.0075 37.8691273513906 -eG 0.05 64.377516497364 -eG 0.075 -3.78691273513906 -eG 0.5 -6.4377516497364 -eG 0.75 0.378691273513906 -eG 5 0.64377516497364 -eG 7.5 -0.0378691273513906 -eG 50 -0.064377516497364 -eG 75 0.00378691273513906 -eN 500 1 -B 1 1 -S 1 σ > fig3.txt

Supplementary Figure 15 :

for σ in 0,0.5,0.8,0.9

./scrm 4 1 -t 5000 -r 25000 1e+07 -eG 5e-04 6437.7516497364 -eG 0.00075 -378.691273513906-eG 0.005 -643.77516497364 -eG 0.0075 37.8691273513906 -eG 0.05 64.377516497364 -eG 0.075 -3.78691273513906 -eG 0.5 -6.4377516497364 -eG 0.75 0.378691273513906 -eG 5 0.64377516497364 -eG 7.5 -0.0378691273513906 -eG 50 -0.064377516497364 -eG 75 0.00378691273513906 -eN 500 1 -B 1 1 -S 1 σ > figs15.txt

Supplementary Figure 16 :

forσ in 0,0.5,0.8,0.9

a):

./scrm 4 1 -t 5000 -r 5000 1e+07 -B 1 1 -S 1 σ > figs16a.txt

b):

./scrm 4 1 -t 5000 -r 5000 1e+07 -eN 0.3 0.1 -eN 2 1 -B 1 1 -S 1 σ > figs16b.txt

c):

./scrm 4 1 -t 5000 -r 5000 1e+07 -eG 0.05 3.576528694298 -eN 0.5 0.2 -B 1 1 -S 1 σ > figs16c.txt

d):

./scrm 4 1 -t 5000 -r 5000 1e+07 -eG 0.05 -3.576528694298 -eN 0.5 5 -B 1 1 -S 1 σ > figs16d.txt

Figure 4 a) :

./scrm 4 1 -t 5000 -r 5000 1e+07 -eG 5e-04 6437.7516497364 -eG 0.00075 -378.691273513906-eG 0.005 -643.77516497364 -eG 0.0075 37.8691273513906 -eG 0.05 64.377516497364 -eG 0.075 -3.78691273513906 -eG 0.5 -6.4377516497364 -eG 0.75 0.378691273513906 -eG 5 0.64377516497364 -eG 7.5 -0.0378691273513906 -eG 50 -0.064377516497364 -eG 75 0.00378691273513906 -eN 500 1 -B 1 0.2 -S 1 0.4 > fig4a.txt

Figure 4 a b) :

./scrm 4 1 -t 5000 -r 5000 1e+07 -eG 5e-04 6437.7516497364 -eG 0.00075 -378.691273513906-eG 0.005 -643.77516497364 -eG 0.0075 37.8691273513906 -eG 0.05 64.377516497364 -eG 0.075 -3.78691273513906 -eG 0.5 -6.4377516497364 -eG 0.75 0.378691273513906 -eG 5 0.64377516497364 -eG 7.5 -0.0378691273513906 -eG 50 -0.064377516497364 -eG 75 0.00378691273513906 -eN 500 1 -B 1 0.6 -S 1 0.857 > fig4b.txt

Figure 4 c) :

./scrm 4 1 -t 5000 -r 5000 1e+07 -eG 5e-04 6437.7516497364 -eG 0.00075 -378.691273513906-eG 0.005 -643.77516497364 -eG 0.0075 37.8691273513906 -eG 0.05 64.377516497364 -eG 0.075 -3.78691273513906 -eG 0.5 -6.4377516497364 -eG 0.75 0.378691273513906 -eG 5 0.64377516497364 -eG 7.5 -0.0378691273513906 -eG 50 -0.064377516497364 -eG 75 0.00378691273513906 -eN 500 1 -B 1 1 -S 1 0.919 > fig4c.txt

Figure 4 d) :

./scrm 4 1 -t 5000 -r 5000 1e+07 -eG 5e-04 6437.7516497364 -eG 0.00075 -378.691273513906-eG 0.005 -643.77516497364 -eG 0.0075 37.8691273513906 -eG 0.05 64.377516497364 -eG 0.075 -3.78691273513906 -eG 0.5 -6.4377516497364 -eG 0.75 0.378691273513906 -eG 5 0.64377516497364 -eG 7.5 -0.0378691273513906 -eG 50 -0.064377516497364 -eG 75 0.00378691273513906 -eN 500 1 -B 1 0.15 -S 1 0 > fig4d.txt

Supplementary Figure 18 a) :

./scrm 4 1 -t 5000 -r 33335 1e+07 -eG 5e-04 6437.7516497364 -eG 0.00075 -378.691273513906-eG 0.005 -643.77516497364 -eG 0.0075 37.8691273513906 -eG 0.05 64.377516497364 -eG 0.075 -3.78691273513906 -eG 0.5 -6.4377516497364 -eG 0.75 0.378691273513906 -eG 5 0.64377516497364 -eG 7.5 -0.0378691273513906 -eG 50 -0.064377516497364 -eG 75 0.00378691273513906 -eN 500 1 -B 1 0.2 -S 1 0.4 > figs18a.txt

Supplementary Figure 18 b) :

./scrm 4 1 -t 5000 -r 33335 1e+07 -eG 5e-04 6437.7516497364 -eG 0.00075 -378.691273513906-eG 0.005 -643.77516497364 -eG 0.0075 37.8691273513906 -eG 0.05 64.377516497364 -eG 0.075 -3.78691273513906 -eG 0.5 -6.4377516497364 -eG 0.75 0.378691273513906 -eG 5 0.64377516497364 -eG 7.5 -0.0378691273513906 -eG 50 -0.064377516497364 -eG 75 0.00378691273513906 -eN 500 1 -B 1 0.6 -S 1 0.857 > figs18b.txt

Supplementary Figure 18 c) :

./scrm 4 1 -t 5000 -r 33335 1e+07 -eG 5e-04 6437.7516497364 -eG 0.00075 -378.691273513906-eG 0.005 -643.77516497364 -eG 0.0075 37.8691273513906 -eG 0.05 64.377516497364 -eG 0.075 -3.78691273513906 -eG 0.5 -6.4377516497364 -eG 0.75 0.378691273513906 -eG 5 0.64377516497364 -eG 7.5 -0.0378691273513906 -eG 50 -0.064377516497364 -eG 75 0.00378691273513906 -eN 500 1 -B 1 1 -S 1 0.919 > figs18c.txt

Supplementary Figure 18 d) :

./scrm 4 1 -t 5000 -r 33335 1e+07 -eG 5e-04 6437.7516497364 -eG 0.00075 -378.691273513906-eG 0.005 -643.77516497364 -eG 0.0075 37.8691273513906 -eG 0.05 64.377516497364 -eG 0.075 -3.78691273513906 -eG 0.5 -6.4377516497364 -eG 0.75 0.378691273513906 -eG 5 0.64377516497364 -eG 7.5 -0.0378691273513906 -eG 50 -0.064377516497364 -eG 75 0.00378691273513906 -eN 500 1 -B 1 0.15 -S 1 0 > figs18d.txt

2 Command lines for analyzing data

Figure 1, Supplementary figure 1,3,4,5,6,7,11 :
eSMC : Fig1 is the Segregating matrix of the 4 Haplotypes.
eSMC(n=40,rho=1,O=Fig1,pop=F,SB=F,SF=F,Rho=T,Check=F)
PSMC': fig1.txt is a multihetsep file of 2 Haplotypes.
./msmc_1.0.0_linux64bit -o f1_output fig1.txt -r 1
MSMC : fig1.txt is a multihetsep file of 4 Haplotypes.
./msmc_1.0.0_linux64bit -o f1_output fig1.txt -r 1
MSMC2 : fig1.txt is a multihetsep file of 4 Haplotypes.
./msmc2_linux64bit -o f1_output fig1.txt -r 1
Supplementary figure 2 :
eSMC : Figs2 is the Segregating matrix of the 4 Haplotypes.
eSMC(n=40,rho=1,O=Figs2,pop=F,SB=F,SF=F,Rho=T,Check=F)
PSMC': figs2.txt is a multihetsep file of 2 Haplotypes.
./msmc_1.0.0_linux64bit -o fs2_output figs2.txt -p 20*2 -r 1
MSMC : figs2.txt is a multihetsep file of 4 Haplotypes.
./msmc_1.0.0_linux64bit -o fs2_output figs2.txt -p 20*2 -r 1
MSMC2 : figs2.txt is a multihetsep file of 4 Haplotypes.

./msmc2_linux64bit -o fs2_output figs2.txt -p 20*2 -r 1
Supplementary figure 8 :
eSMC : Figs8 is the Segregating matrix of the 4 Haplotypes.
eSMC(n=40,rho=5,O=Figs8,pop=F,SB=F,SF=F,Rho=F,Check=F)
PSMC': figs8.txt is a multihetsep file of 2 Haplotypes.
./msmc_1.0.0_linux64bit -o fs8_output figs8.txt –fixedRecombination -r 5
MSMC : figs8.txt is a multihetsep file of 4 Haplotypes.
./msmc_1.0.0_linux64bit -o fs8_output figs8.txt –fixedRecombination -r 5
MSMC2 : figs8.txt is a multihetsep file of 4 Haplotypes.
./msmc2_linux64bit -o fs8_output figs8.txt –fixedRecombination -r 5
Supplementary figure 9 :
eSMC : Figs9 is the Segregating matrix of the 4 Haplotypes.
eSMC(n=40,rho=100,O=Figs9,pop=F,SB=F,SF=F,Rho=F,Check=F)
PSMC': figs9.txt is a multihetsep file of 2 Haplotypes.
./msmc_1.0.0_linux64bit -o fs9_output figs9.txt –fixedRecombination -r 100
MSMC : figs9.txt is a multihetsep file of 4 Haplotypes.
./msmc_1.0.0_linux64bit -o fs9_output figs9.txt –fixedRecombination -r 100
MSMC2 : figs9.txt is a multihetsep file of 4 Haplotypes.
./msmc2_linux64bit -o fs9_output figs9.txt –fixedRecombination -r 100
Supplementary figure 10 :
eSMC : Figs10 is the Segregating matrix of the 4 Haplotypes.
eSMC(n=40,rho=5,O=Figs10,pop=F,SB=F,SF=F,Rho=T,Check=F)
PSMC': figs10.txt is a multihetsep file of 2 Haplotypes.
./msmc_1.0.0_linux64bit -o fs10_output figs10.txt -r 5
MSMC : figs10.txt is a multihetsep file of 4 Haplotypes.
./msmc_1.0.0_linux64bit -o fs10_output figs10.txt -r 5
MSMC2 : figs8.txt is a multihetsep file of 4 Haplotypes.
./msmc2_linux64bit -o fs10_output figs10.txt -r 5
Figure 2, Supplementary figure 12,13,14 :
eSMC : Fig2 is the Segregating matrix of the 4 Haplotypes.
eSMC(n=40,rho=1,O=Fig2,pop=F,SB=T,SF=F,Rho=F,Check=F)
PSMC': fig2.txt is a multihetsep file of 2 Haplotypes.
./msmc_1.0.0_linux64bit -o f2_output fig2.txt -r 1
MSMC : fig2.txt is a multihetsep file of 4 Haplotypes.
./msmc_1.0.0_linux64bit -o f2_output fig2.txt -r 1
MSMC2 : fig2.txt is a multihetsep file of 4 Haplotypes.
./msmc2_linux64bit -o f2_output fig2.txt -r 1
Figure 3, Supplementary figure 16 :
eSMC : Fig3 is the Segregating matrix of the 4 Haplotypes.
eSMC(n=40,rho=1,O=Fig3,pop=F,SB=F,SF=T,Rho=F,Check=F)
PSMC': fig3.txt is a multihetsep file of 2 Haplotypes.
./msmc_1.0.0_linux64bit -o f3_output fig3.txt -r 1
MSMC : fig3.txt is a multihetsep file of 4 Haplotypes.
./msmc_1.0.0_linux64bit -o f3_output fig3.txt -r 1
MSMC2 : fig3.txt is a multihetsep file of 4 Haplotypes.
./msmc2_linux64bit -o f3_output fig3.txt -r 1
Supplementary figure 15 :
eSMC : Figs15 is the Segregating matrix of the 4 Haplotypes.
eSMC(n=40,rho=5,O=Figs15,pop=F,SB=F,SF=T,Rho=F,Check=F)
PSMC': figs15.txt is a multihetsep file of 2 Haplotypes.
./msmc_1.0.0_linux64bit -o fs15_output figs15.txt -r 1
MSMC : figs15.txt is a multihetsep file of 4 Haplotypes.
./msmc_1.0.0_linux64bit -o fs15_output figs15.txt -r 1
MSMC2 : figs15.txt is a multihetsep file of 4 Haplotypes.
./msmc2_linux64bit -o fs15_output figs15.txt -r 1

Figure 4 a) :
eSMC : Fig4a is the Segregating matrix of the 4 Haplotypes.
eSMC(n=40,rho=1,O=Fig4a,pop=F,SB=F,SF=F,Rho=T,Check=F)
eSMC(n=40,rho=1,O=Fig4a,pop=F,SB=T,SF=T,Rho=F,Check=F)
eSMC(n=40,rho=1,O=Fig4a,pop=F,SB=T,SF=T,Rho=F,Check=F,Boxs=c(0,0.5))
eSMC(n=40,rho=1,O=Fig4a,pop=F,SB=T,SF=T,Rho=F,Check=F,BoxB=c(0.05,0.5))
eSMC(n=40,rho=1,O=Fig4a,pop=F,SB=T,SF=T,Rho=F,Check=F,Boxs=c(0,0.5),BoxB=c(0.05,0.5))
Figure 4 b) :
eSMC : Fig4b is the Segregating matrix of the 4 Haplotypes.
eSMC(n=40,rho=1,O=Fig4b,pop=F,SB=F,SF=F,Rho=T,Check=F)
eSMC(n=40,rho=1,O=Fig4b,pop=F,SB=T,SF=T,Rho=F,Check=F)
eSMC(n=40,rho=1,O=Fig4b,pop=F,SB=T,SF=T,Rho=F,Check=F,Boxs=c(0.5,0.99))
eSMC(n=40,rho=1,O=Fig4b,pop=F,SB=T,SF=T,Rho=F,Check=F,BoxB=c(0.5,1))
eSMC(n=40,rho=1,O=Fig4b,pop=F,SB=T,SF=T,Rho=F,Check=F,Boxs=c(0.5,0.99),BoxB=c(0.5,1))
Figure 4 c) :
eSMC : Fig4c is the Segregating matrix of the 4 Haplotypes.
eSMC(n=40,rho=1,O=Fig4c,pop=F,SB=F,SF=F,Rho=T,Check=F)
eSMC(n=40,rho=1,O=Fig4c,pop=F,SB=T,SF=T,Rho=F,Check=F)
eSMC(n=40,rho=1,O=Fig4c,pop=F,SB=T,SF=T,Rho=F,Check=F,Boxs=c(0.5,0.99))
eSMC(n=40,rho=1,O=Fig4c,pop=F,SB=T,SF=T,Rho=F,Check=F,BoxB=c(0.5,1))
eSMC(n=40,rho=1,O=Fig4c,pop=F,SB=T,SF=T,Rho=F,Check=F,Boxs=c(0.5,0.99),BoxB=c(0.5,1))
Figure 4 d) :
eSMC : Fig4d is the Segregating matrix of the 4 Haplotypes.
eSMC(n=40,rho=1,O=Fig4d,pop=F,SB=F,SF=F,Rho=T,Check=F)
eSMC(n=40,rho=1,O=Fig4d,pop=F,SB=T,SF=T,Rho=F,Check=F)
eSMC(n=40,rho=1,O=Fig4d,pop=F,SB=T,SF=T,Rho=F,Check=F,Boxs=c(0,0.5))
eSMC(n=40,rho=1,O=Fig4d,pop=F,SB=T,SF=T,Rho=F,Check=F,BoxB=c(0.05,0.5))
eSMC(n=40,rho=1,O=Fig4d,pop=F,SB=T,SF=T,Rho=F,Check=F,Boxs=c(0,0.5),BoxB=c(0.05,0.5))
Supplementary figure 18 a) :
eSMC : Figs18a is the Segregating matrix of the 4 Haplotypes.
eSMC(n=40,rho=6.667,O=Figs18a,pop=F,SB=F,SF=F,Rho=T,Check=F)
eSMC(n=40,rho=6.667,O=Figs18a,pop=F,SB=T,SF=T,Rho=F,Check=F)
eSMC(n=40,rho=6.667,O=Figs18a,pop=F,SB=T,SF=T,Rho=F,Check=F,Boxs=c(0,0.5))
eSMC(n=40,rho=6.667,O=Figs18a,pop=F,SB=T,SF=T,Rho=F,Check=F,BoxB=c(0.05,0.5))
eSMC(n=40,rho=6.667,O=Figs18a,pop=F,SB=T,SF=T,Rho=F,Check=F,Boxs=c(0,0.5),BoxB=c(0.05,0.5))
Supplementary figure 18 b) :
eSMC : Figs18b is the Segregating matrix of the 4 Haplotypes.
eSMC(n=40,rho=6.667,O=Figs18b,pop=F,SB=F,SF=F,Rho=T,Check=F)
eSMC(n=40,rho=6.667,O=Figs18b,pop=F,SB=T,SF=T,Rho=F,Check=F)
eSMC(n=40,rho=6.667,O=Figs18b,pop=F,SB=T,SF=T,Rho=F,Check=F,Boxs=c(0.5,0.99))
eSMC(n=40,rho=6.667,O=Figs18b,pop=F,SB=T,SF=T,Rho=F,Check=F,BoxB=c(0.5,1))
eSMC(n=40,rho=6.667,O=Figs18b,pop=F,SB=T,SF=T,Rho=F,Check=F,Boxs=c(0.5,0.99),BoxB=c(0.5,1))
Supplementary figure 18 c) :
eSMC : Figs18c is the Segregating matrix of the 4 Haplotypes.
eSMC(n=40,rho=6.667,O=Figs18c,pop=F,SB=F,SF=F,Rho=T,Check=F)
eSMC(n=40,rho=6.667,O=Figs18c,pop=F,SB=T,SF=T,Rho=F,Check=F)
eSMC(n=40,rho=6.667,O=Figs18c,pop=F,SB=T,SF=T,Rho=F,Check=F,Boxs=c(0.5,0.99))
eSMC(n=40,rho=6.667,O=Figs18c,pop=F,SB=T,SF=T,Rho=F,Check=F,BoxB=c(0.5,1))
eSMC(n=40,rho=6.667,O=Figs18c,pop=F,SB=T,SF=T,Rho=F,Check=F,Boxs=c(0.5,0.99),BoxB=c(0.5,1))
Supplementary figure 18 d) :
eSMC : Figs18d is the Segregating matrix of the 4 Haplotypes.
eSMC(n=40,rho=6.667,O=Figs18d,pop=F,SB=F,SF=F,Rho=T,Check=F)
eSMC(n=40,rho=6.667,O=Figs18d,pop=F,SB=T,SF=T,Rho=F,Check=F)
eSMC(n=40,rho=6.667,O=Figs18d,pop=F,SB=T,SF=T,Rho=F,Check=F,Boxs=c(0,0.5))
eSMC(n=40,rho=6.667,O=Figs18d,pop=F,SB=T,SF=T,Rho=F,Check=F,BoxB=c(0.05,0.5))

eSMC(n=40,rho=6.667,O=Figs18d,pop=F,SB=T,SF=T,Rho=F,Check=F,Boxs=c(0,0.5),BoxB=c(0.05,0.5))

Figure 5 : $rho_chr = 4.857143, 5.142857, 5.000000, 5.428571, 5.142857$

For all five chromosome (noted chr)

a)

$eSMC(n = 40, rho = rho\_chr[chr], O = Fig5, pop = F, SB = F, SF = T, Rho = F, Check = F)$

b)

$eSMC(n = 40, rho = rho\_chr[chr], O = Fig5, pop = F, SB = T, SF = T, Rho = F, Check = F, Boxs = c(0.5, 0.99), BoxB = c(0.5, 1))$

Supplementary figure 20 :

eSMC : Figs20 is the Segregating matrix of the 6 individuals.

eSMC(n=40,rho=1,O=Figs20,pop=F,SB=F,SF=F,Rho=T,Check=F)

PSMC': figs20.txt is a multihetsep file of 2 Haplotypes.

./msmc_1.0.0_linux64bit -o fs20_output figs20.txt -r 1

MSMC : figs20.txt is a multihetsep file of the 6 individuals.

./msmc_1.0.0_linux64bit -o fs20_output figs20.txt -r 1

MSMC2 : figs20.txt is a multihetsep file of the 6 individuals.

./msmc2_linux64bit -o fs20_output figs20.txt -r 1

Figure 6 :

eSMC : Fig6 is the Segregating matrix of 1 individual.

eSMC(n=40,rho=18.47575,O=Fig6,pop=F,SB=T,SF=F,Rho=F,Check=F)

eSMC(n=40,rho=9.237875,O=Fig6,pop=F,SB=T,SF=F,Rho=F,Check=F)

eSMC(n=40,rho=3.69515,O=Fig6,pop=F,SB=T,SF=F,Rho=F,Check=F)

PSMC': fig6.txt is a multihetsep file of 1 individual.

./msmc_1.0.0_linux64bit -o f6_output fig6.txt -r 1
