# Supplementary figures and images for: Correction: Inference of past demography, dormancy and self-fertilization rates from whole genome sequence data
Source: PLoS Genet. 2021 Apr 7;17(4):e1009504. doi: 10.1371/journal.pgen.1009504 (PMC8026070; doi:10.1371/journal.pgen.1009504)

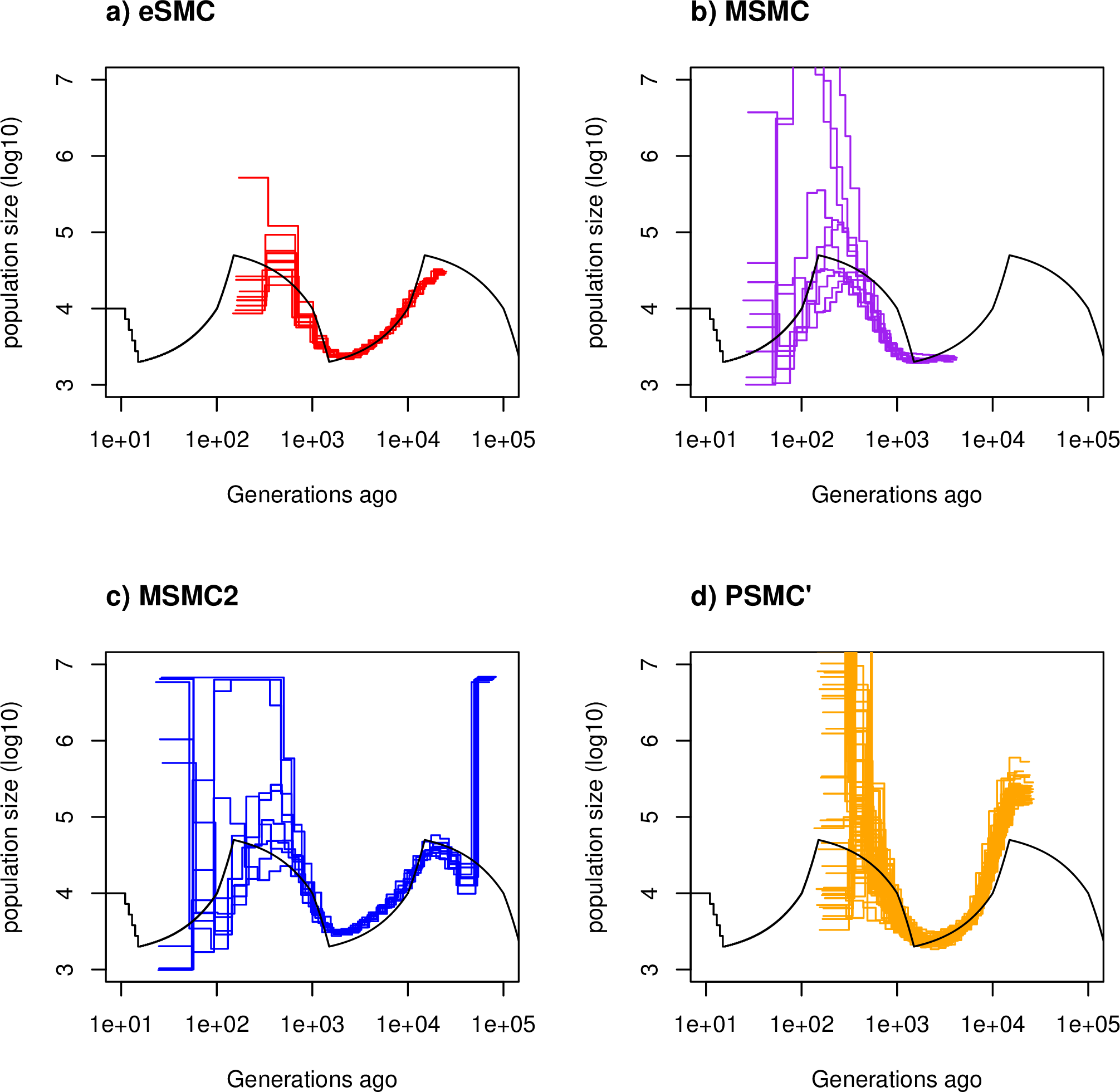

Supplement: S1 Fig — Estimated demographic history using four simulated sequences of 10 Mb under a saw-tooth demographic scenario with 10 replicates. Mutation and recombination rate are set to 1.25 × 10^−8^ per generation per bp. Therefore ρθ=rμ=1. The simulated demographic history is represented in black. a) Demographic history estimated by eSMC (red). b) Demographic history estimated by MSMC (purple). c) Demographic history estimated by MSMC2 (blue). d) Demographic history estimated by PSMC’ (orange). (TIF) [file pgen.1009504.s002.tif]

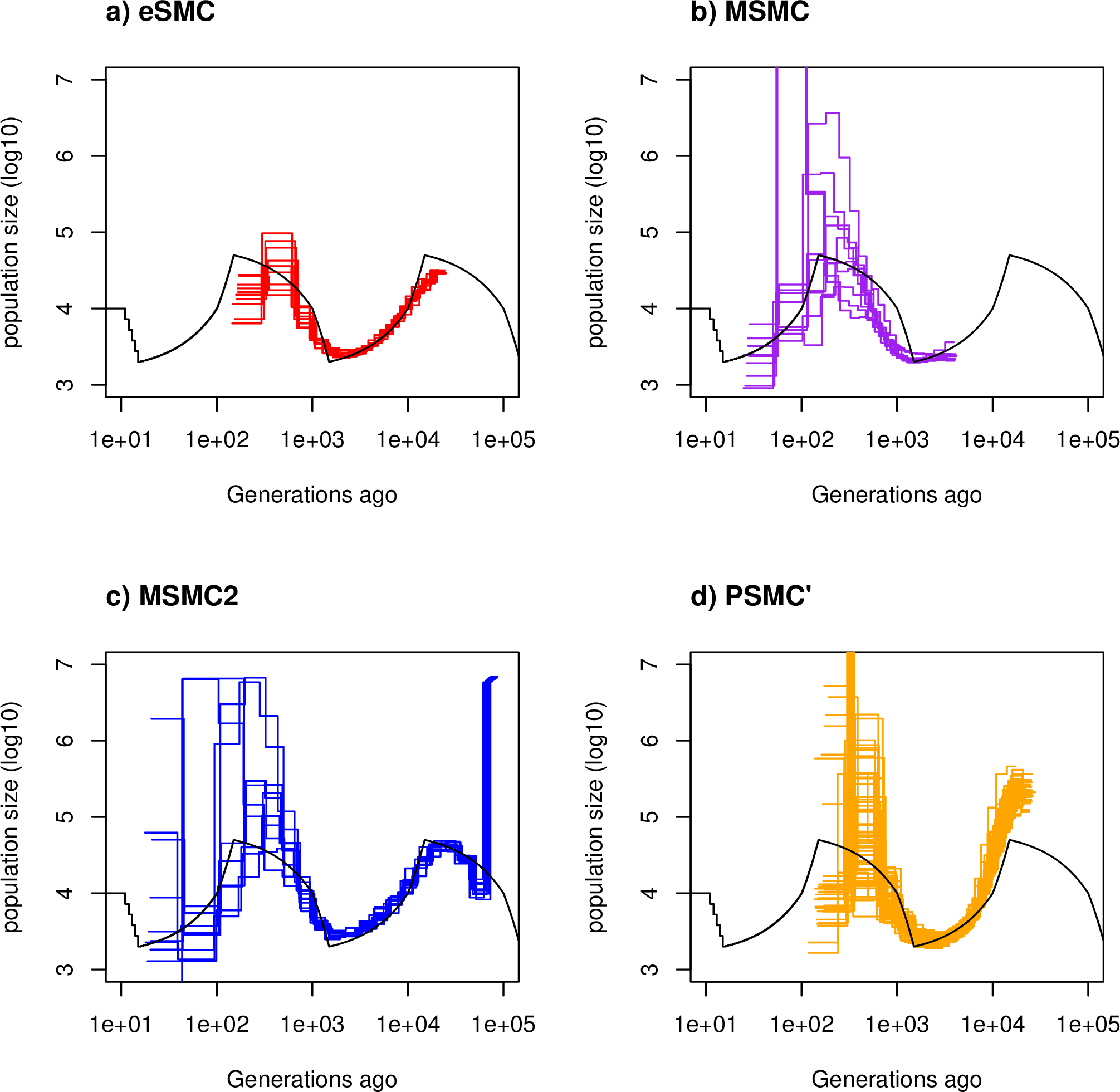

Supplement: S2 Fig — Estimated demographic history using four simulated sequences of 10 Mb under a sawtooth demographic scenario with 10 replicates. Mutation and recombination rate are set to 1.25 × 10^−8^ per generation per bp. Therefore ρθ=rμ=1. The simulated demographic history is represented in black. a) Demographic history estimated by eSMC (red). b) Demographic history estimated by MSMC (purple). c) Demographic history estimated by MSMC2 (blue). d) Demographic history estimated by PSMC’ (orange). (TIF) [file pgen.1009504.s003.tif]

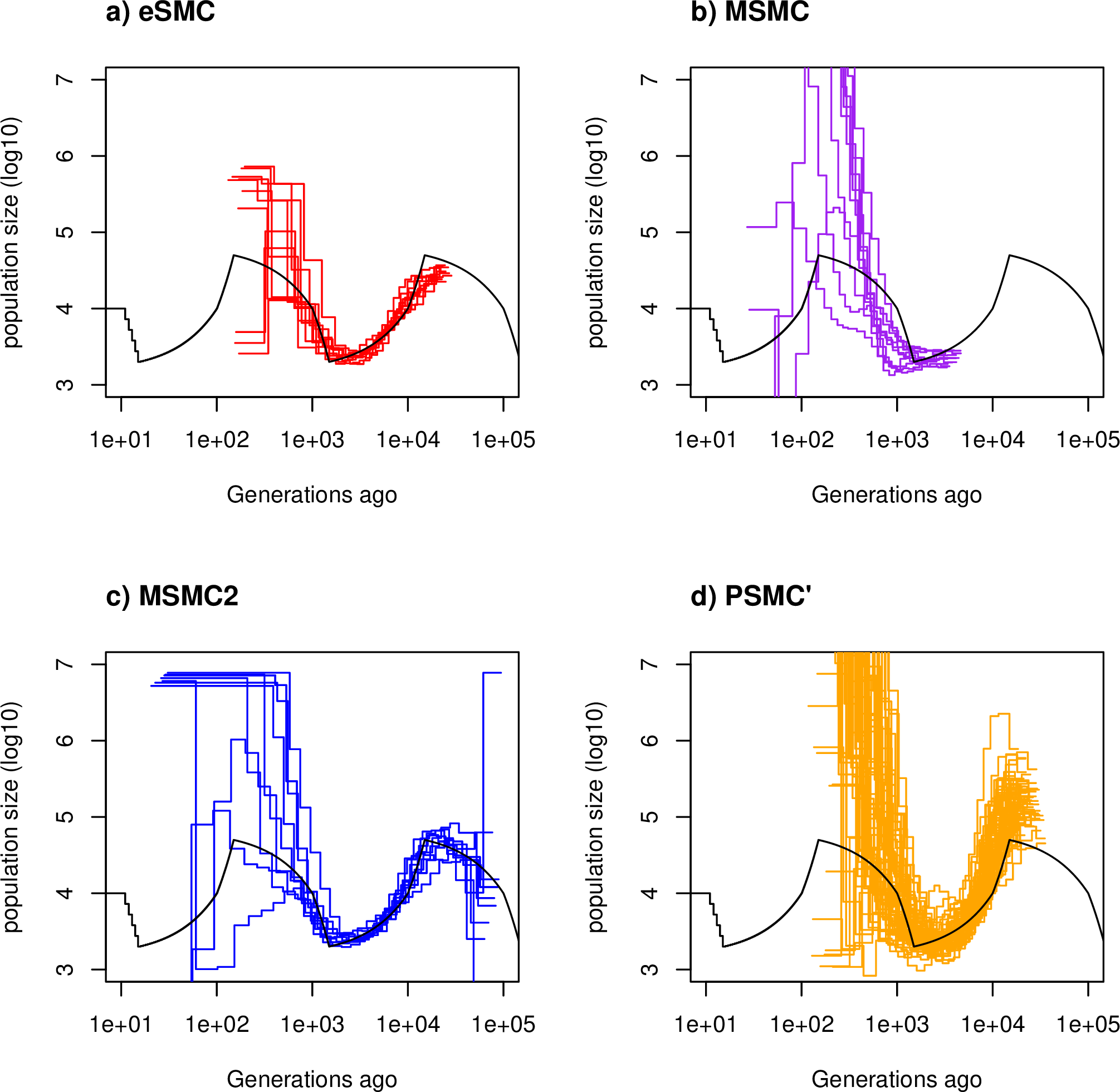

Supplement: S3 Fig — Estimated demographic history using four simulated sequences of 1 Mb under a saw-tooth scenario with 10 replicates. Mutation and recombination rate are set to 1.25 × 10^−8^ per generation per bp. Therefore ρθ=rμ=1. The simulated demographic history is represented in black. a) Demographic history estimated by eSMC (red). b) Demographic history estimated by MSMC (purple). c) Demographic history estimated by MSMC2 (blue). d) Demographic history estimated by PSMC’ (orange). (TIF) [file pgen.1009504.s004.tif]

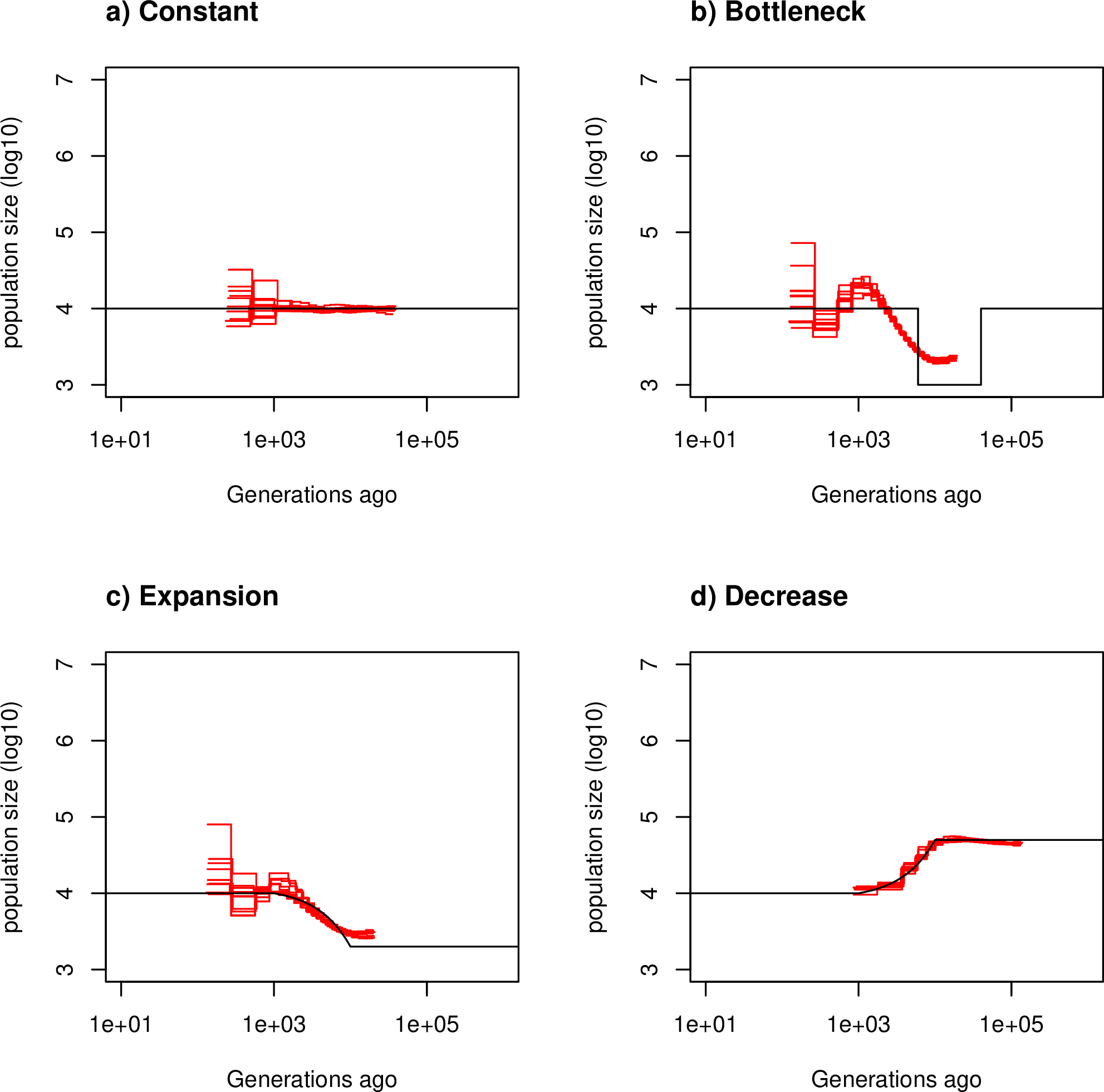

Supplement: S4 Fig — Estimated demographic history using four simulated sequences of 10 Mb under 4 different demographic scenarios with 10 replicates. Mutation and recombination rate are set to 1.25 × 10^−8^ per generation per bp. Therefore ρθ=rμ=1. The simulated demographic history is represented in black. a) Demographic history simulated under a constant population size. b) Demographic history simulated under a bottleneck. c) Demographic history simulated under an expansion. d) Demographic history simulated under a decrease. Demographic history estimated by eSMC is in red. (TIF) [file pgen.1009504.s005.tif]

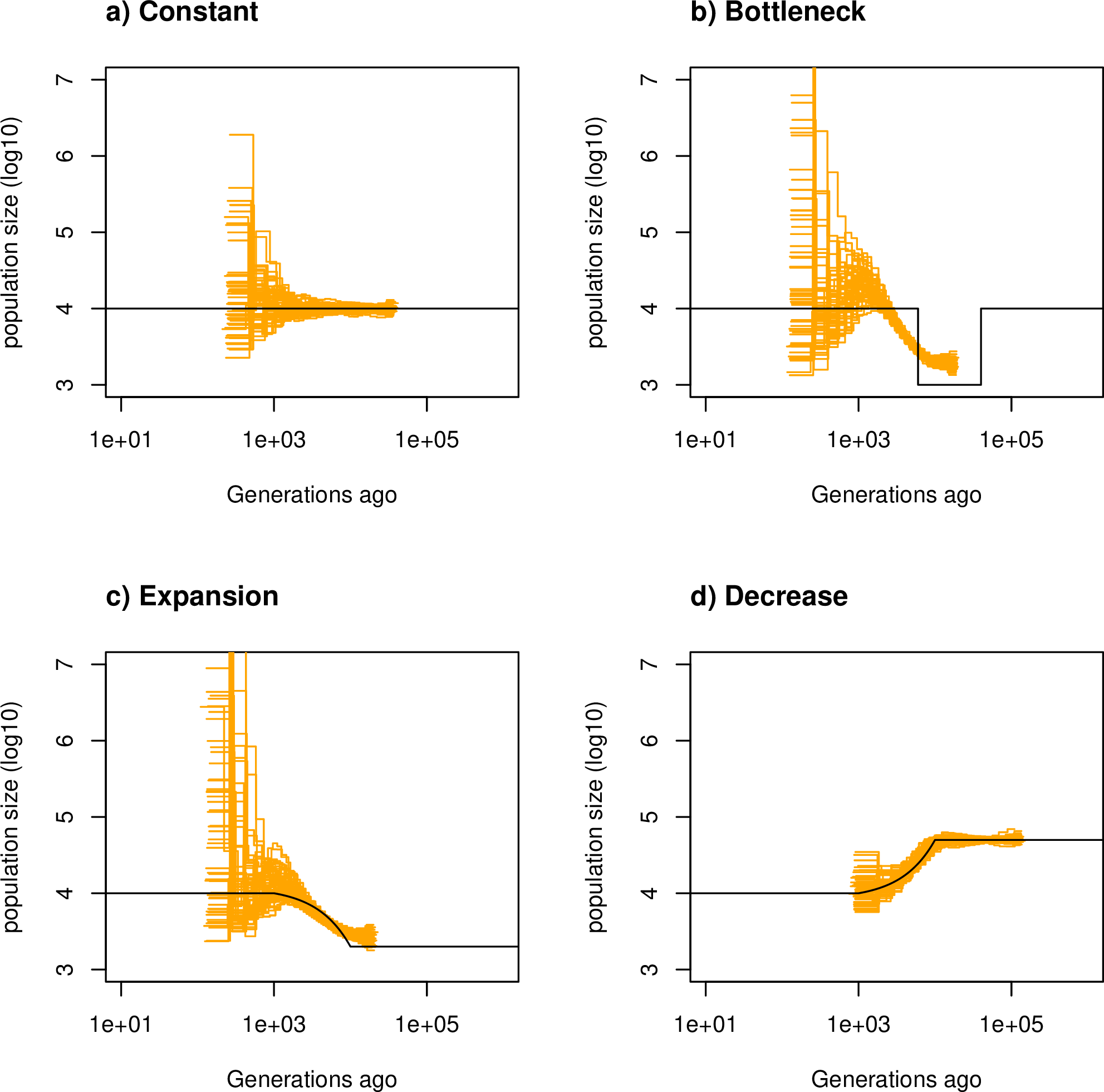

Supplement: S5 Fig — Estimated demographic history using four simulated sequences of 10 Mb under 4 different demographic scenarios with 10 replicates. Mutation and recombination rate are set to 1.25 × 10^−8^ per generation per bp. Therefore ρθ=rμ=1. The simulated demographic history is represented in black. a) Demographic history simulated under a constant population size. b) Demographic history simulated under a bottleneck. c) Demographic history simulated under an expansion. d) Demographic history simulated under a decrease. Demographic history estimated by PSMC’ is in orange. (TIF) [file pgen.1009504.s006.tif]

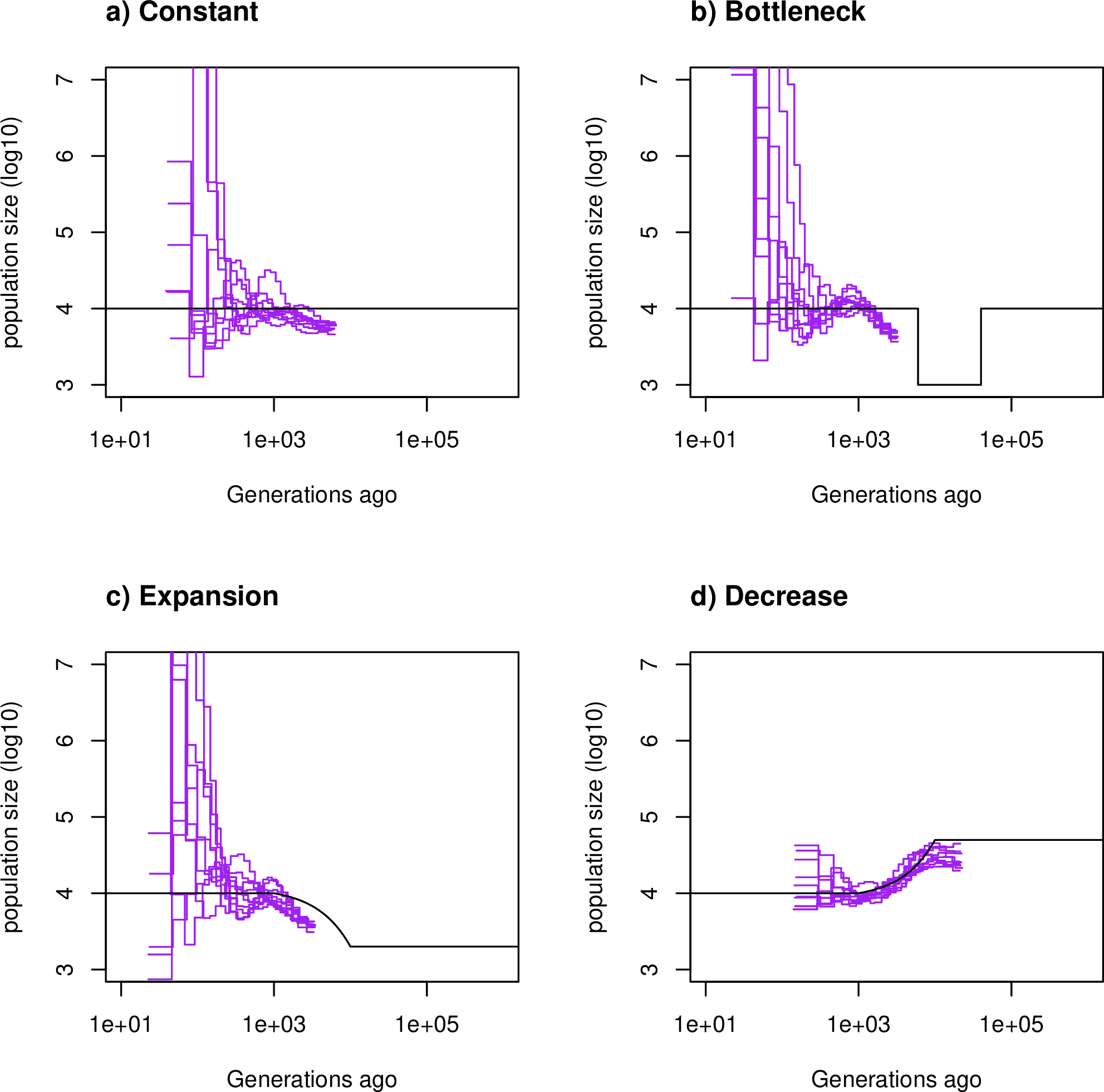

Supplement: S6 Fig — Estimated demographic history using four simulated sequences of 10 Mb under 4 different demographic scenarios with 10 replicates. Mutation and recombination rate are set to 1.25 × 10^−8^ per generation per bp. Therefore ρθ=rμ=1. The simulated demographic history is represented in black. a) Demographic history simulated under a constant population size. b) Demographic history simulated under a bottleneck. c) Demographic history simulated under an expansion. d) Demographic history simulated under a decrease. Demographic history estimated by MSMC is in purple. (TIF) [file pgen.1009504.s007.tif]

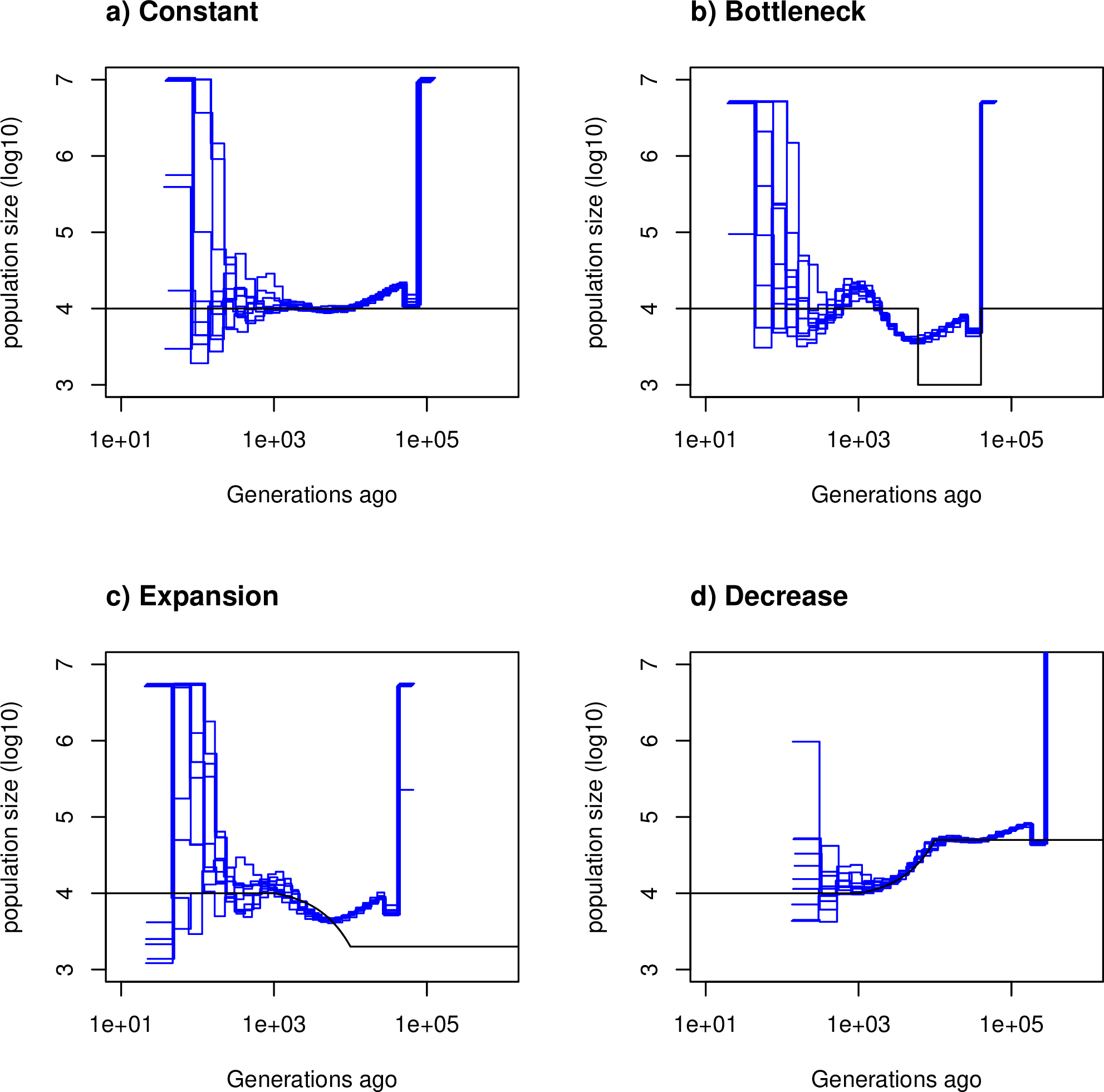

Supplement: S7 Fig — Estimated demographic history using four simulated sequences of 10 Mb under 4 different demographic scenarios with 10 replicates. Mutation and recombination rate are set to 1.25 × 10^−8^ per generation per bp. Therefore ρθ=rμ=1. The simulated demographic history is represented in black. a) Demographic history simulated under a constant population size. b) Demographic history simulated under a bottleneck. c) Demographic history simulated under an expansion. d) Demographic history simulated under a decrease. Demographic history estimated by MSMC2 is in blue. (TIF) [file pgen.1009504.s008.tif]

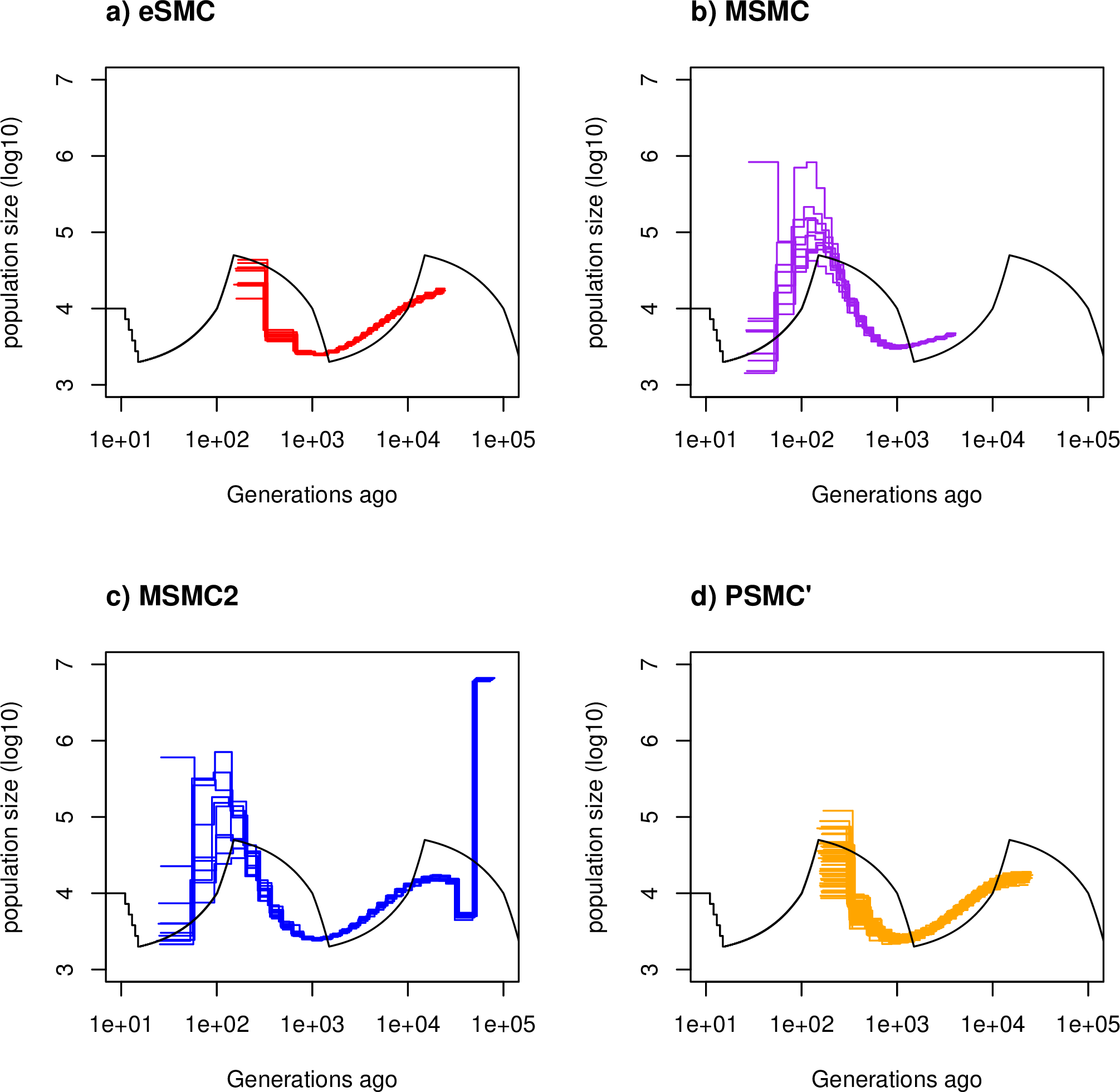

Supplement: S8 Fig — Results are obtained by fixing recombination rate to real value. Estimated demographic history using four simulated sequences of 10 Mb under a saw-tooth scenario with 10 replicates. Mutation and recombination rate are set to 1.25 × 10^−8^ and 6.25 × 10^−8^ per generation per bp. Therefore ρθ=rμ=5. The simulated demographic history is represented in black. a) Demographic history estimated by eSMC (red). b) Demographic history estimated by MSMC (purple). c) Demographic history estimated by MSMC2 (blue). d) Demographic history estimated by PSMC’ (orange). (TIF) [file pgen.1009504.s009.tif]

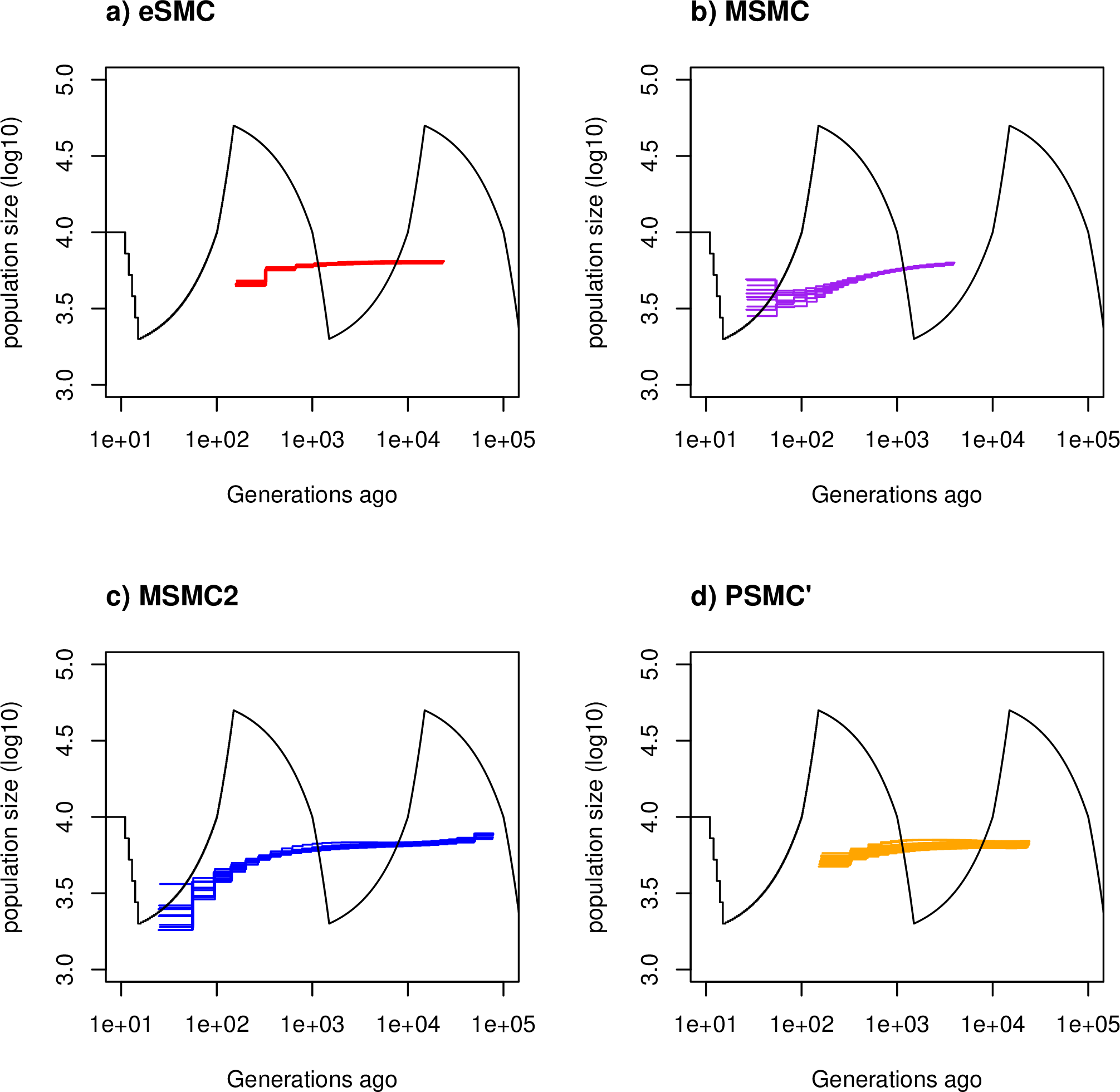

Supplement: S9 Fig — Results are obtained by fixing recombination rate to real value. Estimated demographic history using four simulated sequences of 10 Mb under a saw-tooth scenario with 10 replicates. Mutation and recombination rate are set to 1.25 × 10^−8^ and 1.25 × 10^−6^ per generation per bp. Therefore ρθ=rμ=100. The simulated demographic history is represented in black. a) Demographic history estimated by eSMC (red). b) Demographic history estimated by MSMC (purple). c) Demographic history estimated by MSMC2 (blue). d) Demographic history estimated by PSMC’ (orange). (TIF) [file pgen.1009504.s010.tif]

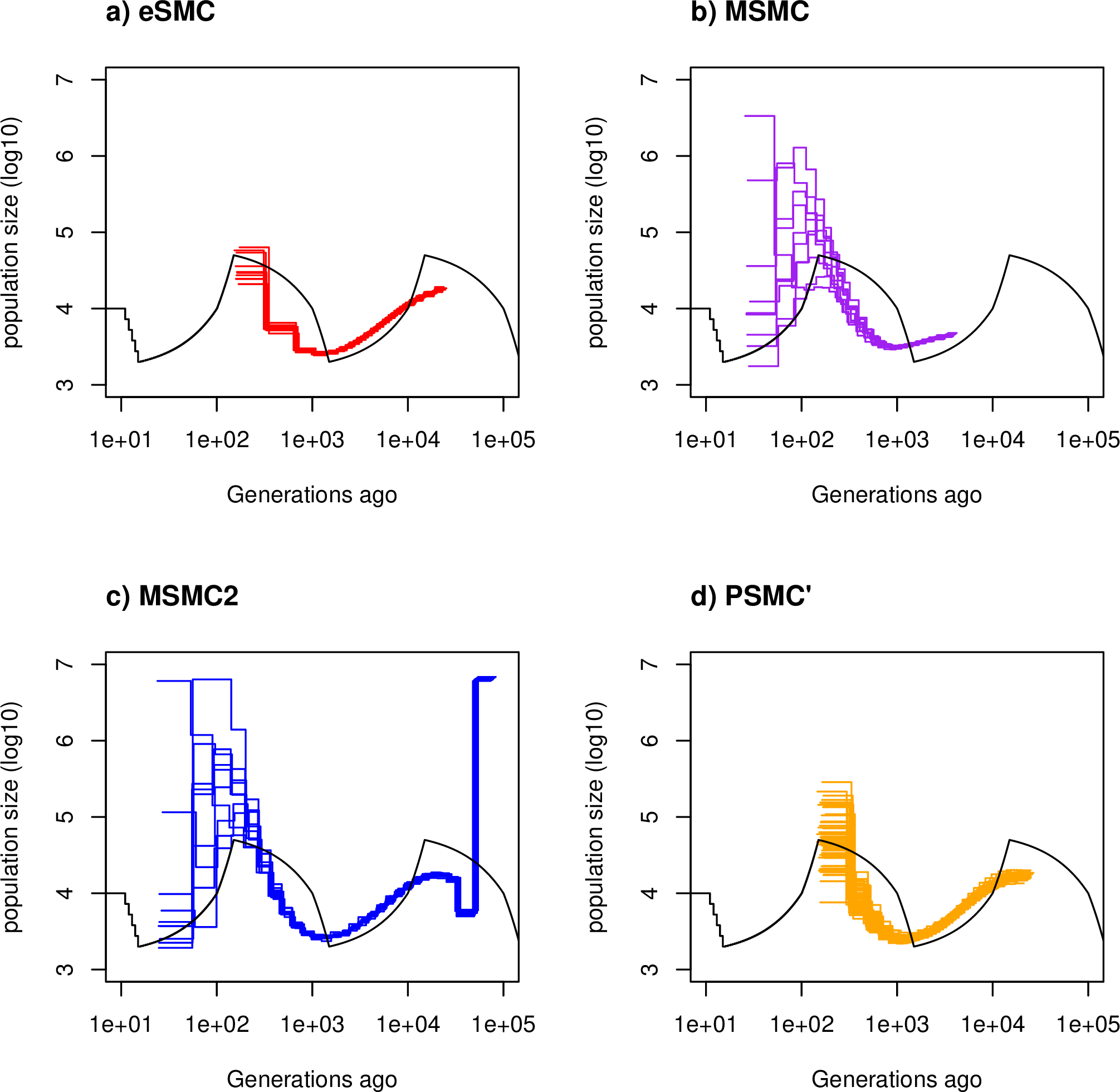

Supplement: S10 Fig — Results are obtained by estimating recombination rate with initial value equal to mutation rate (ρθ=5). Estimated demographic history using four simulated sequences of 10 Mb under a saw-tooth scenario with 10 replicates. Mutation and recombination rate are set to 1.25 × 10^−8^ and 6.25 × 10^−8^ per generation per bp. Therefore ρθ=rμ=5. The simulated demographic history is represented in black. a) Demographic history estimated by eSMC (red). b) Demographic history estimated by MSMC (purple). c) Demographic history estimated by MSMC2 (blue). d) Demographic history estimated by PSMC’ (orange). (TIF) [file pgen.1009504.s011.tif]

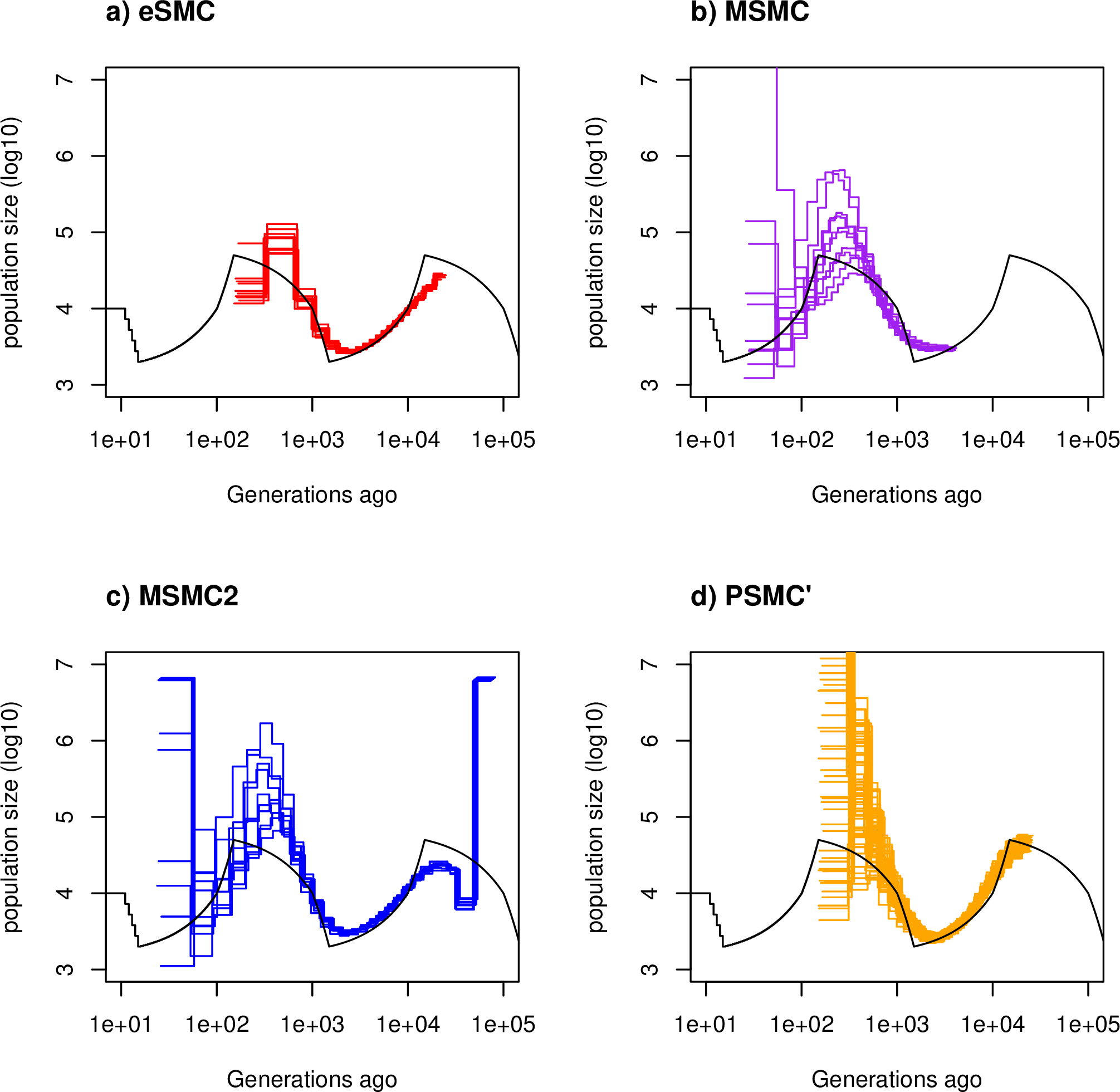

Supplement: S11 Fig — Results are obtained by estimating recombination rate with initial value equal to mutation rate (ρθ=1). Estimated demographic history using four simulated sequences of 10 Mb under a saw-tooth scenario with 10 replicates. Mutation and recombination rate are set to 1.25 × 10^−8^ and 6.25 × 10^−8^ per generation per bp. Therefore ρθ=rμ=5. The simulated demographic history is represented in black. a) Demographic history estimated by eSMC (red). b) Demographic history estimated by MSMC (purple). c) Demographic history estimated by MSMC2 (blue). d) Demographic history estimated by PSMC’ (orange). (TIF) [file pgen.1009504.s012.tif]

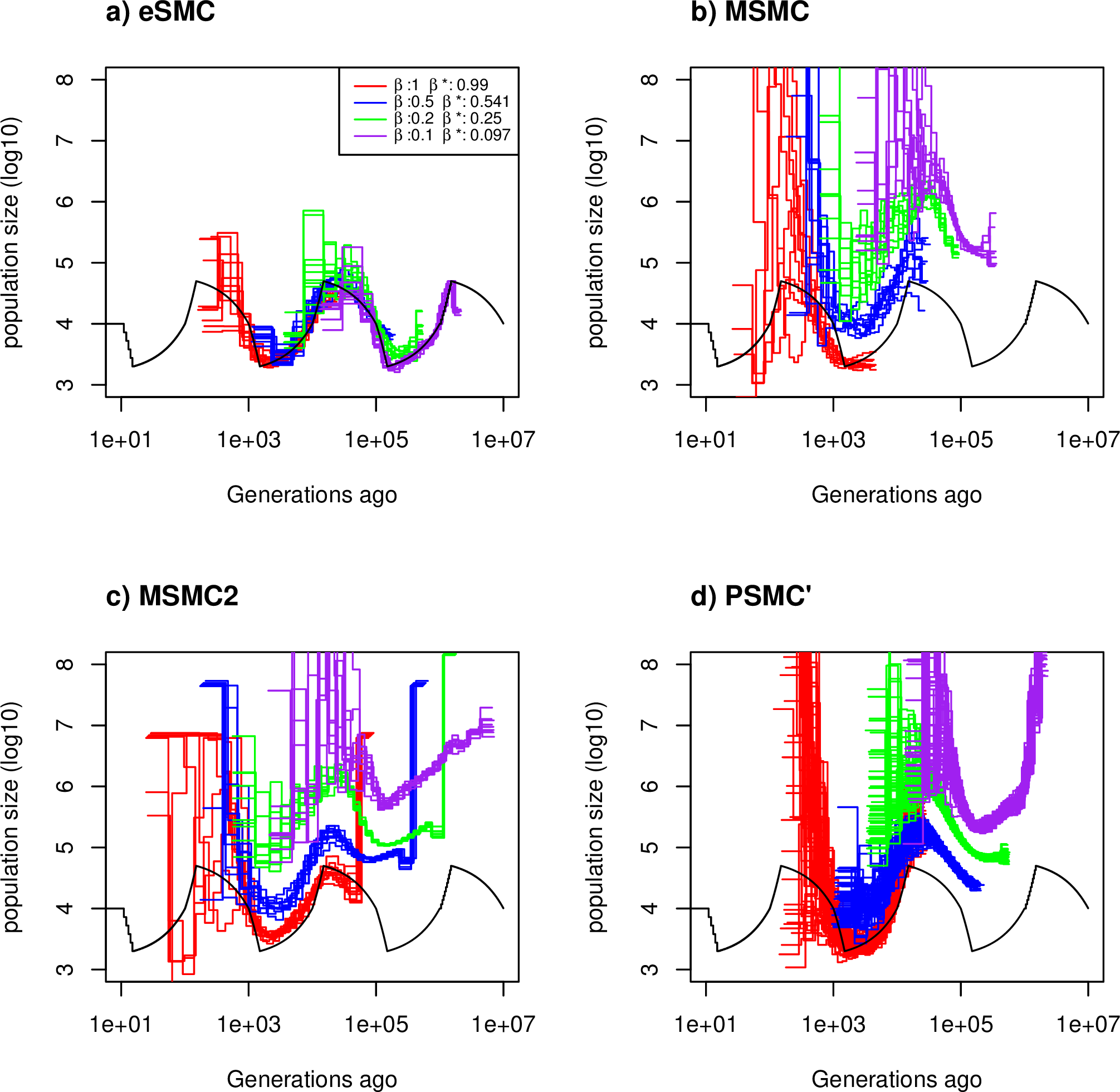

Supplement: S12 Fig — Estimated demographic history using four simulated sequences of 10 Mb and ten replicates under a sawtooth demographic scenario (black). Simulation were done under four different germination rate β (1,0.5,0.2 and 0.1). The mutation and recombination rates are set to 2.5 × 10^−9^ per generation per bp. Therefore rμ=1 and respectively ρθ=1, ρθ=0.5, ρθ=0.2 and ρθ=0.1. Estimated demographic history are represented for all tested germination rate, β = 1 (red), 0.5 (blue), 0.2 (green) and 0.1 (purple). The demographic history is estimated using a) eSMC where β* equal the estimated germination rate, b) MSMC, c) MSMC2 and d) PSMC’. (TIF) [file pgen.1009504.s013.tif]

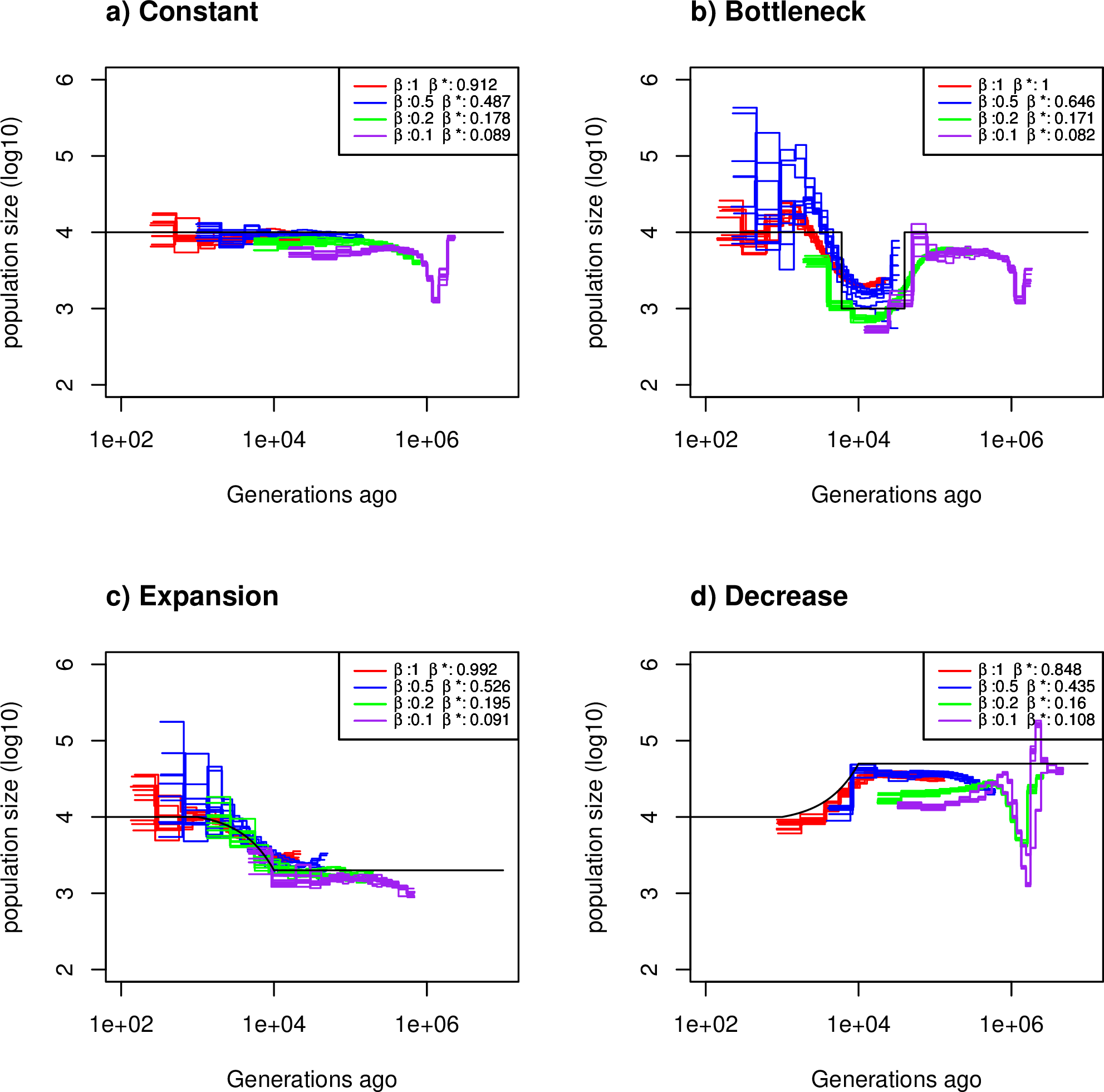

Supplement: S13 Fig — Estimated demographic history using four simulated sequences of 10 Mb under four different demographic scenarios with 10 replicates. Mutation and recombination rate are set to 1.25 × 10^−8^ per generation per bp. Simulation were done under four different germination rates β. We have β = 1 (red), 0.5 (blue), 0.2 (green) and 0.1 (purple). Therefore rμ=1 and respectively ρθ=1, ρθ=0.5, ρθ=0.2 and ρθ=0.1. The simulated demographic history is represented in black. a) Demographic history simulated under a constant population size. b) Demographic history simulated under a bottleneck. c) Demographic history simulated under an expansion. d) Demographic history simulated under a decrease. In addition we simulated data under four different germination rate β. β* equal the estimated germination rate. (TIF) [file pgen.1009504.s014.tif]

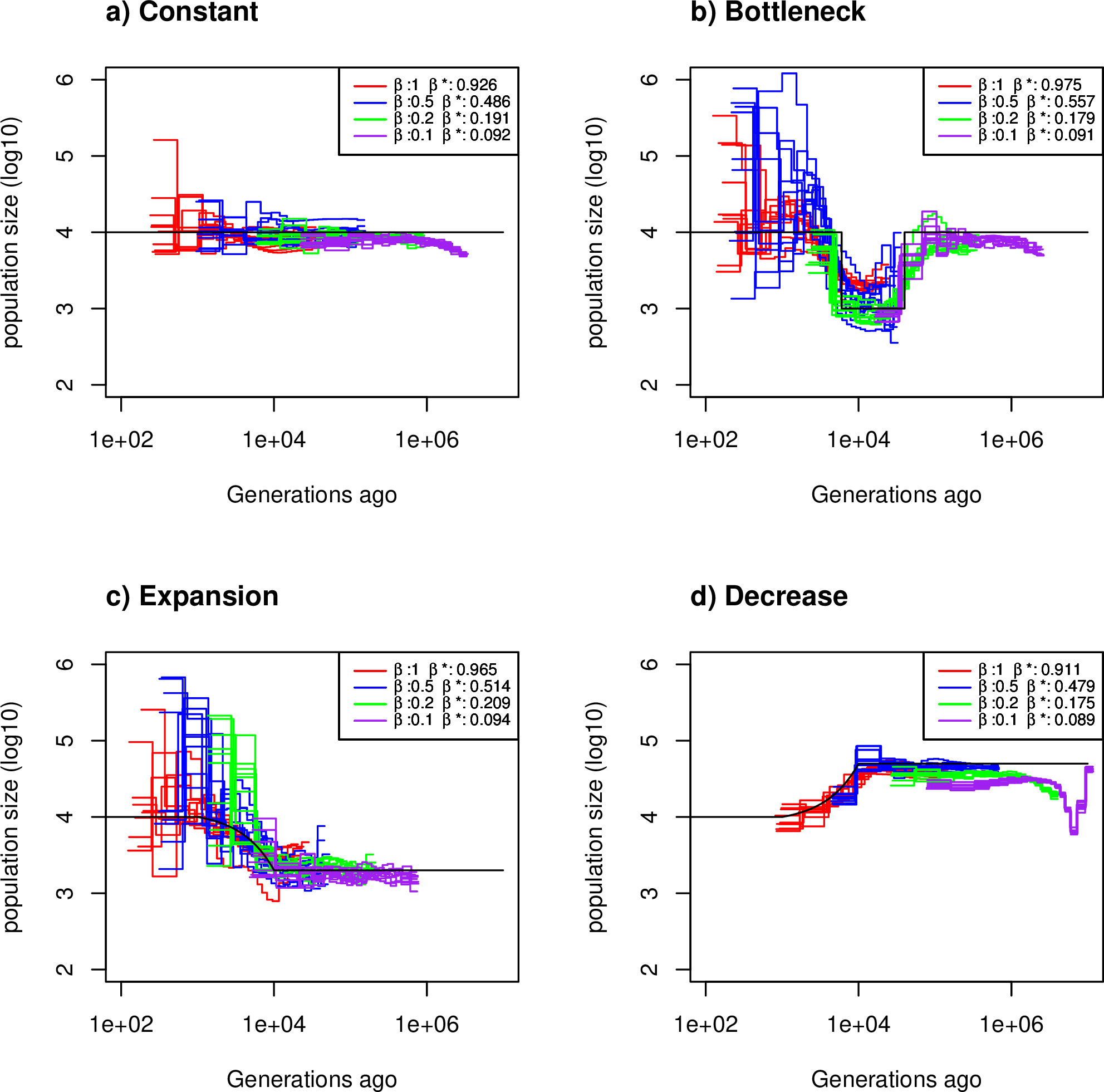

Supplement: S14 Fig — Estimated demographic history using four simulated sequences of 10 Mb under four different demographic scenarios with 10 replicates. Mutation and recombination rate are set to 2.5 × 10^−9^ per generation per bp. Simulation were done under four different germination rate b. We have β = 1 (red), 0.5 (blue), 0.2 (green) and 0.1 (purple). Therefore rμ=1 and respectively ρθ=1, ρθ=0.5, ρθ=0.2 and ρθ=0.1. The simulated demographic history is represented in black. a) Demographic history simulated under a constant population size. b) Demographic history simulated under a bottleneck. c) Demographic history simulated under an expansion. d) Demographic history simulated under a decrease. In addition we simulated data under four different germination rate β. β* equal the estimated germination rate. (TIF) [file pgen.1009504.s015.tif]

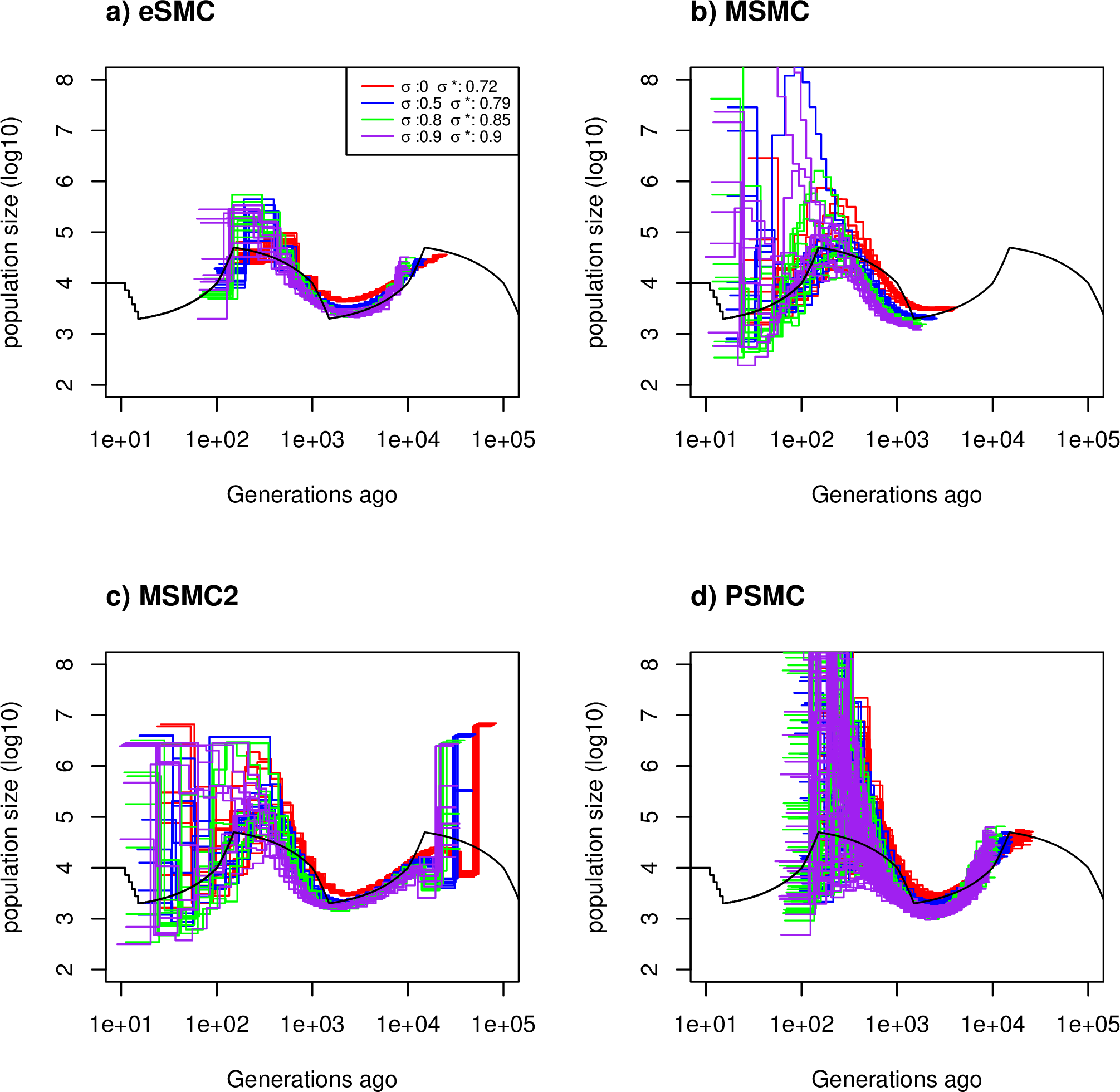

Supplement: S15 Fig — Estimated demographic history using four simulated sequences of 10 Mb and ten replicates under a saw-tooth demographic scenario (black). Simulation were done under four different self-fertilization rate σ (0,0.5,0.8 and 0.9). The mutation is set to 1.25 × 10^−8^ and the recombination rate to 6.25 × 10^−8^ per generation per bp. Therefore rμ=5 and respectively ρθ=5, ρθ=3.335, ρθ=1.667 and ρθ=0.91. Estimated demographic history are represented for all tested self-fertilization, σ = 1 (red), 0.5 (blue), 0.2 (green) and 0.1 (purple). The demographic history is estimated using a) eSMC where σ* equals the estimated self-fertilization rate, b) MSMC, c) MSMC2 and d) PSMC’. (TIF) [file pgen.1009504.s016.tif]

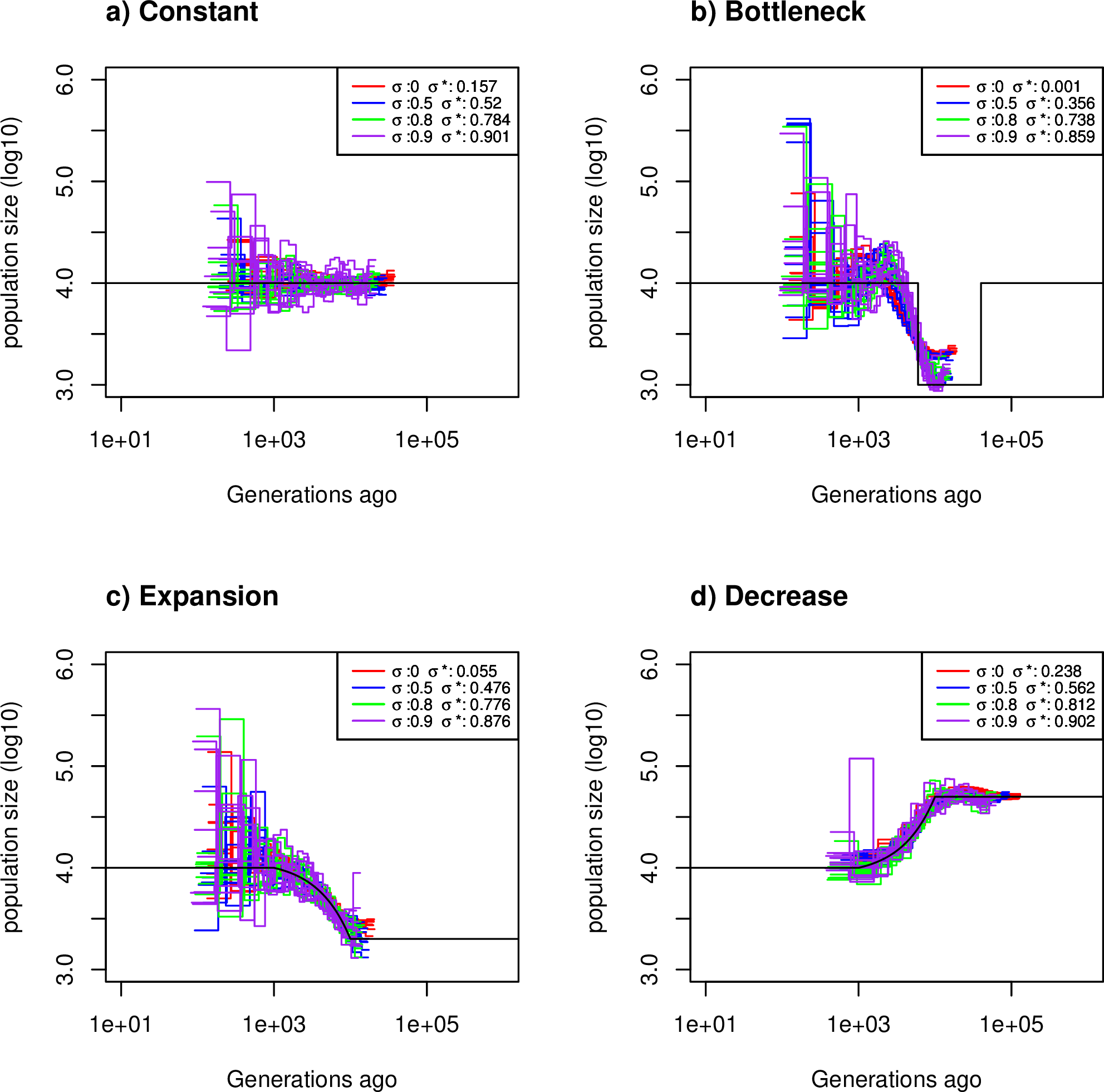

Supplement: S16 Fig — Estimated demographic history using four simulated sequences of 10 Mb under four different demographic scenarios with 10 replicates. Mutation and recombination rate are set to 1.25 × 10^−8^ per generation per bp. Simulation were done under four different self-fertilization rate σ (0,0.5,0.8 and 0.9). Therefore rμ=1 and respectively ρθ=1, ρθ=0.667, ρθ=0.333 and ρθ=0.182. The simulated demographic history is represented in black. a) Demographic history simulated under a constant population size. b) Demographic history simulated under a bottleneck. c) Demographic history simulated under an expansion. d) Demographic history simulated under a decrease. In addition we simulated data under four different self-fertilization rate σ. We have σ = 0 (red), 0.5 (blue), 0.8 (green) and 0.9 (purple). σ* equal the estimated self-fertilization rate. (TIF) [file pgen.1009504.s017.tif]

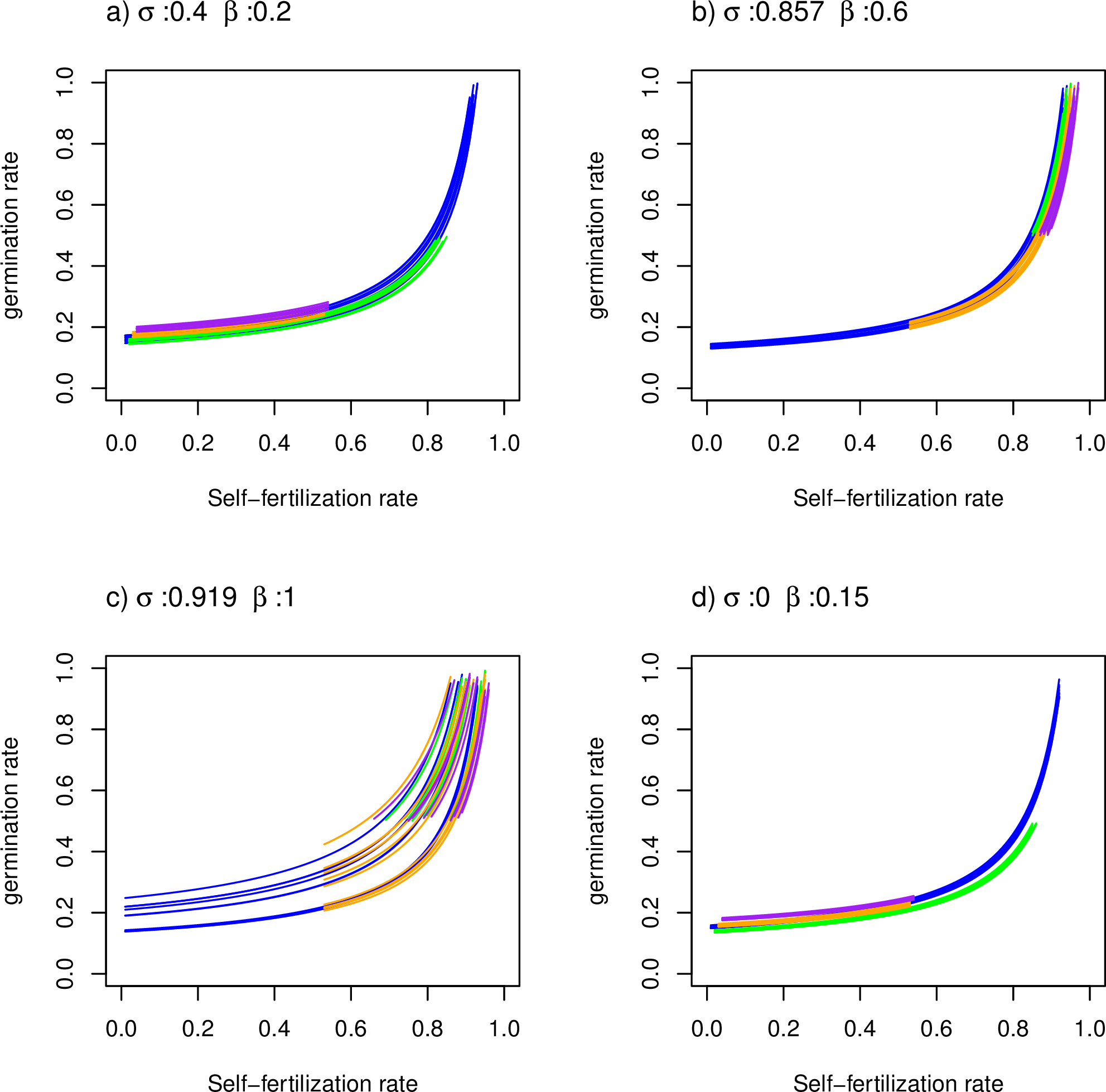

Supplement: S17 Fig — Possible estimated self-fertilization and germination rates because of confounding effect using four simulated sequences of 10 Mb under a saw-tooth demographic scenario and four different combinations of germination (b) and self-fertilization (s) rate but resulting in the same ρθ=0.15. Mutation rate is set to 1.25 × 10^−8^ and recombination rate to 1.25 × 10^−8^ per generation per bp. Therefore rμ=1. The four combination are: a) σ = 0.4 and β = 0.2, b) σ = 0.857 and β = 0.6, c) σ = 0.919 and β = 1 and d) σ = 0 and β = 0.15. Hence, for each scenario ρθ=0.15. For each combination of β and σ, eSMC was launched with five different prior settings: ignoring seed banks and self-fertilization (red), accounting for seed banks and self-fertilization but without setting priors (blue), accounting for seed banks and self-fertilization with a prior set only for the self-fertilization rate (green), only for the germination rate (orange) or for both (purple). (TIF) [file pgen.1009504.s018.tif]

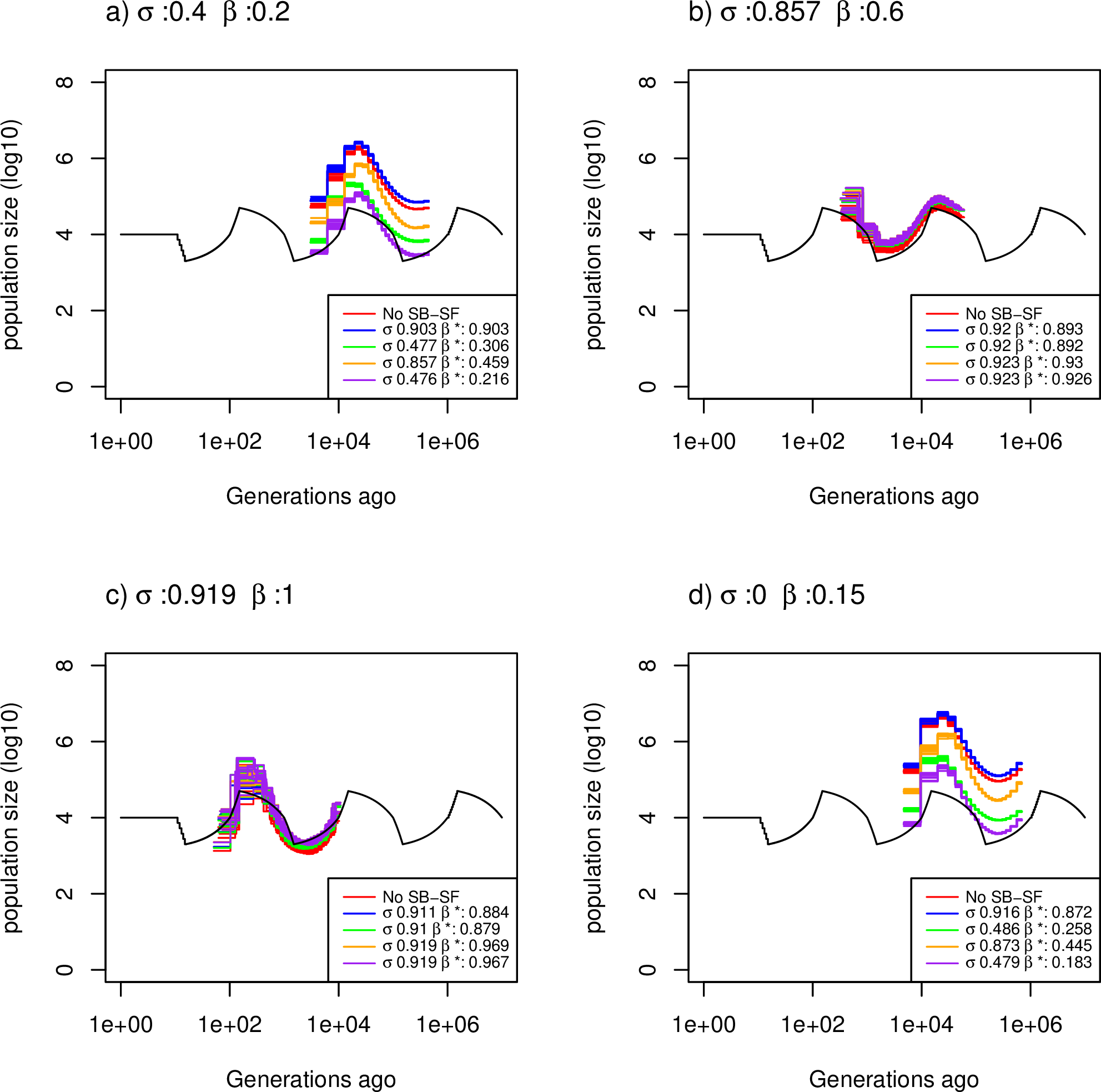

Supplement: S18 Fig — Demographic history estimated by eSMC for ten replicates using four simulated sequences of 10 Mb under a saw-tooth demographic scenario and four different combinations of germination (b) and self-fertilization (s) rate but resulting in the same ρθ=1. Mutation rate is set to 1.25 × 10^−8^ and recombination rate to 8.335 × 10^−8^ per generation per bp. Therefore rμ=6.67. The four combination are: a) σ = 0.4 and β = 0.25, b) σ = 0.75 and β = 0.6, c) σ = 0.85 and β = 1 and d) σ = 0 and β = 0.15. Hence, for each scenario ρθ=1. For each combination of β and σ, eSMC was launched with five different prior settings: ignoring seed banks and self-fertilization (red), accounting for seed banks and self-fertilization but without setting priors (blue), accounting for seed banks and self-fertilization with a prior set only for the self-fertilization rate (green), only for the germination rate (orange) or for both (purple). σ* and β* respectively represent the estimated self-fertilization and germination rate. (TIF) [file pgen.1009504.s019.tif]

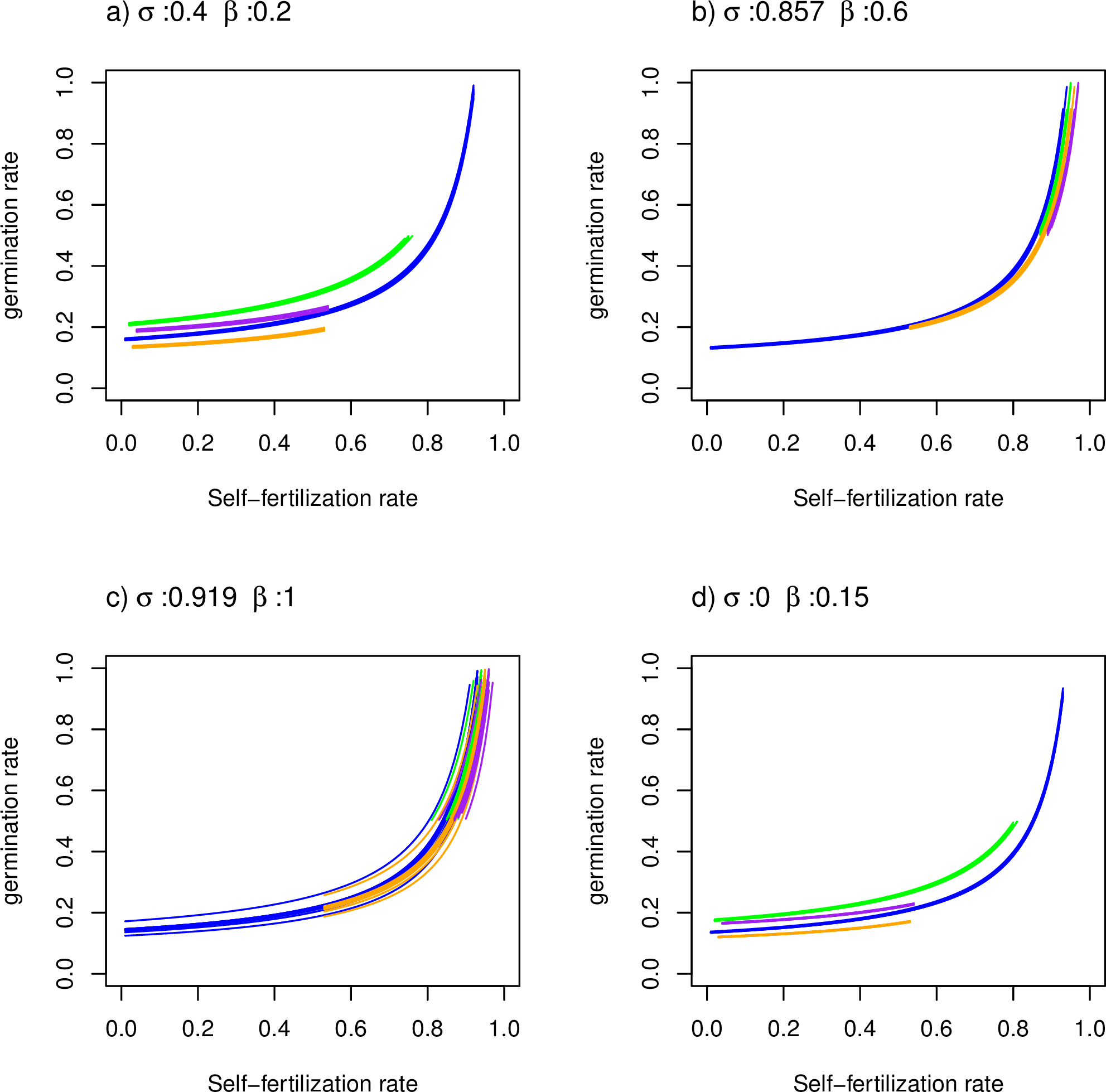

Supplement: S19 Fig — Possible estimated self-fertilization and germination rates because of confounding effect using four simulated sequences of 10 Mb under a saw-tooth demographic scenario and four different combinations of germination (b) and self-fertilization (s) rate but resulting in the same ρθ=1. Mutation rate is set to 1.25 × 10^−8^ and recombination rate to 8.335 × 10^−7^ per generation per bp. Therefore rμ=6.667. The four combination are: a) σ = 0.4 and β = 0.2, b) σ = 0.857 and β = 0.6, c) σ = 0.919 and β = 1 and d) σ = 0 and β = 0.15. Hence, for each scenario ρθ=1 For each combination of β and σ, eSMC was launched with five different prior settings: ignoring seed banks and self-fertilization (red), accounting for seed banks and self-fertilization but without setting priors (blue), accounting for seed banks and self-fertilization with a prior set only for the self-fertilization rate (green), only for the germination rate (orange) or for both (purple). (TIF) [file pgen.1009504.s020.tif]

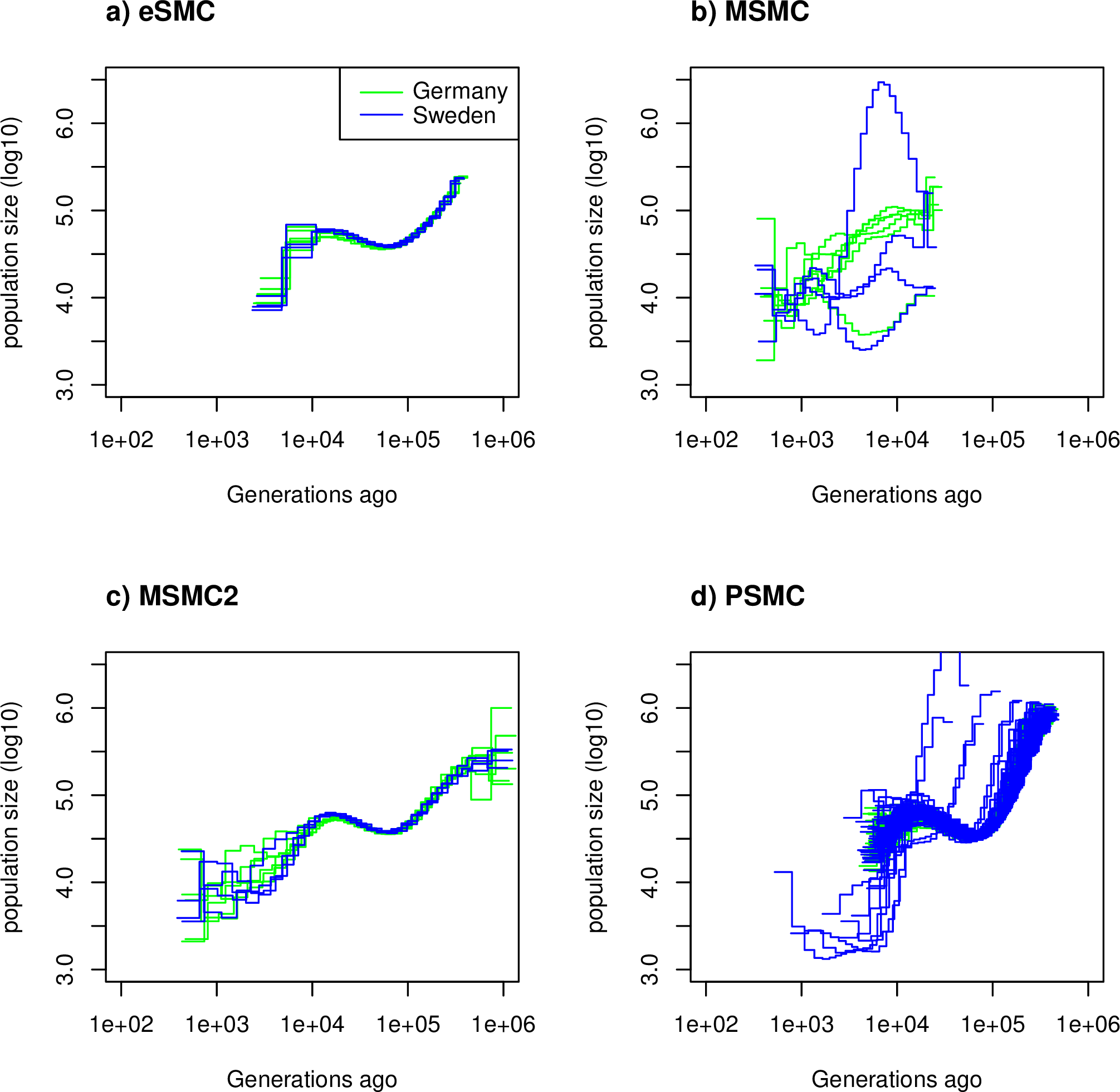

Supplement: S20 Fig — Demographic history of two European (Sweden (blue) and German (green)) populations of A. thaliana. Mutation rate is set to 7 × 10^−9^ per generation per bp and was use as prior for recombination rate. a) Demographic history estimated by eSMC without accounting self-fertilzation or dormancy. b) Demographic history estimated by MSMC. c) Demographic history estimated by MSMC2. d) Demographic history estimated by PSMC’. (TIF) [file pgen.1009504.s021.tif]
